# Maximizing Active Fe Species in ZSM-5 Zeolite Using Organic-Template-Free Synthesis for Efficient Selective Methane Oxidation

**DOI:** 10.1021/jacs.2c13351

**Published:** 2023-02-14

**Authors:** Qingpeng Cheng, Guanna Li, Xueli Yao, Lirong Zheng, Junhu Wang, Abdul-Hamid Emwas, Pedro Castaño, Javier Ruiz-Martínez, Yu Han

**Affiliations:** † Physical Sciences and Engineering Division, Advanced Membranes and Porous Materials (AMPM) Center, King Abdullah University of Science and Technology (KAUST), Thuwal 23955-6900, Saudi Arabia; ‡ KAUST Catalysis Center (KCC), KAUST, Thuwal 23955-6900, Saudi Arabia; § Biobased Chemistry and Technology, Wageningen University & Research, Bornse Weilanden 9, Wageningen 6708WG, The Netherlands; ∥ Laboratory of Organic Chemistry, Wageningen University & Research, Stippeneng 4, Wageningen 6708WE, The Netherlands; ⊥ Beijing Synchrotron Radiation Facility, Institute of High Energy Physics, Chinese Academy of Sciences, Beijing 100049 China; # Center for Advanced Mössbauer Spectroscopy, Dalian Institute of Chemical Physics, Chinese Academy of Sciences, Dalian, Liaoning 116023 China; ¶ Imaging and Characterization Core Lab, King Abdullah University of Science and Technology, Thuwal 23955-6900, Saudi Arabia

## Abstract

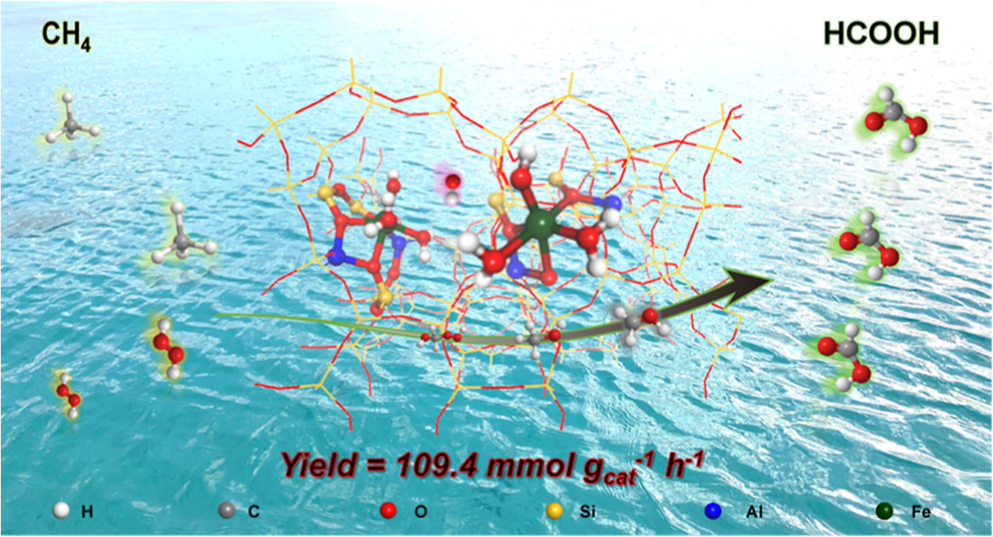

The selective oxidation of CH_4_ in the aqueous phase to produce valuable chemicals has attracted considerable attention due to its mild reaction conditions and simple process. As the most widely studied catalyst for this reaction, Fe-ZSM-5 demonstrates high intrinsic activity and selectivity; however, Fe-ZSM-5 prepared using conventional methods has a limited number of active Fe sites, resulting in low CH_4_ conversion per unit mass of the catalyst. This study reports a facile organic-template-free synthesis strategy that enables the incorporation of more Fe into the zeolite framework with a higher dispersion degree compared to conventional synthesis methods. Because framework Fe incorporated in this way is more readily transformed into isolated extra-framework Fe species under thermal treatment, the overall effect is that Fe-ZSM-5 prepared using this method (Fe-HZ5-TF) has 3 times as many catalytically active sites as conventional Fe-ZSM-5. When used for the selective oxidation of CH_4_ with 0.5 M H_2_O_2_ at 75 °C, Fe-HZ5-TF produced a high C_1_ oxygenate yield of 109.4 mmol g_cat_^–1^ h^–1^ (a HCOOH selectivity of 91.1%), surpassing other catalysts reported to date. Spectroscopic characterization and density functional theory calculations revealed that the active sites in Fe-HZ5-TF are mononuclear Fe species in the form of [(H_2_O)_3_Fe(IV)=O]^2+^ bound to Al pairs in the zeolite framework. This differs from conventional Fe-ZSM-5, where binuclear Fe acts as the active site. Analysis of the catalyst and product evolution during the reaction suggests a radical-driven pathway to explain CH_4_ activation at the mononuclear Fe site and subsequent conversion to C_1_ oxygenates.

## Introduction

The catalytic conversion of CH_4_ to valuable chemicals and clean fuel is an important strategy for efficiently utilizing natural gas, gas hydrates, and shale gas.^1^ The conventional routes for CH_4_ conversion include steam reforming,^2,3^ dry reforming,^4,5^ oxidative coupling,^6,7^ and dehydroaromatization,^8,9^ which often require harsh reaction conditions to activate the highly stable C–H bonds. Selective oxidation is another compelling route for CH_4_ conversion that directly produces multiple oxygenated compounds, such as methanol,^10,11^ formic acid,^12,13^ and acetic acid,^14,15^ under relatively mild conditions. The selective oxidation of CH_4_ with molecular oxygen (O_2_)^16,17^ or other inexpensive oxidants (e.g., N_2_O^16^ and H_2_O^18^) has been demonstrated in gas-phase reactions using zeolite-supported Cu catalysts and in liquid-phase reactions using zeolite-supported noble metals (e.g., Rh,^14^ Ir,^19^ and Au/Pd^10^) catalysts. However, these systems generally exhibit limited CH_4_ conversion, leading to low oxygenate yields.

Non-noble metal iron (Fe)-based catalysts can selectively convert CH_4_ in water when H_2_O_2_ is used as a desirable benign oxidant.^13,20,21^ Of these catalysts, zeolite Fe-ZSM-5 has been the most widely studied.^22−26^ Conventional Fe-ZSM-5 is synthesized by adding an Fe precursor to a synthetic gel of ZSM-5 containing tetrapropylammonium (TPA), which acts as an organic structure-directing agent (also referred to as the template). After synthesis, Fe-ZSM-5 is ion-exchanged and calcined to remove the organic template and generate acidity. During calcination, certain Fe atoms migrate out of the zeolite framework and transform into extra-framework Fe species, taking the forms of Fe-oxo (Fe_*x*_O_*y*_) clusters in the micropores or oxide particles on the external surface of zeolites.^12,26,27^

Despite the coexistence of multiple Fe species in Fe-ZSM-5 and the lack of evidence for their respective roles, the extra-framework binuclear Fe species are generally considered to be the active sites for the selective oxidation of CH_4_,^12,26−29^ possibly because the well-known enzyme soluble CH_4_ monooxygenase (sMMO) contains a di-iron center as the active site.^23^ Combining the results of X-ray absorption spectroscopy and theoretical calculations, Hammond et al. concluded that the binuclear Fe species in Fe-ZSM-5 is [Fe_2_(μ_2_-OH)_2_(OH)_2_(H_2_O)_2_]^2+^ bound to two ion-exchange (Al) sites in the zeolite framework.^26−28^ They proposed that, following the addition of H_2_O_2_, the two Fe centers synergistically activate CH_4_, with Fe(IV)=O extracting an H atom from CH_4_ and Fe–OOH capturing the produced methyl radical.^26^ The idea of constructing binuclear Fe sites to mimic the reactivity of sMMO has been implemented in a metal–organic framework system.^21^ However, other studies have demonstrated that Fe does not necessarily need to take a binuclear form to activate CH_4_. For example, individual Fe atoms anchored on graphene are highly active in this reaction.^20^ Single-atom catalysts based on Pd,^30^ Cr,^31^ and Rh^32^ also play a role in the selective oxidation of CH_4_ at low temperatures, confirming that binuclear metal sites are not indispensable.

A major limitation of conventional Fe-ZSM-5 systems is the low density of catalytically active sites. As a doping element with a large atomic radius, the maximum amount of Fe that can be incorporated into the ZSM-5 framework via direct synthesis is only ∼1.5 wt %.^29,33^ In addition, only a small proportion of framework Fe can be converted into extra-framework Fe, of which only a fraction can act as active sites. Therefore, for conventional Fe-ZSM-5, despite the high intrinsic activity of active Fe species, their low density results in limited CH_4_ conversion per unit mass of the catalyst. Although the Fe loading can be increased using postsynthesis methods, such as ion exchange and impregnation, most Fe species introduced in this way are in the form of oligomeric clusters and bulk oxide particles,^13,25−27^ which are inactive or detrimental to the reaction by promoting nonselective H_2_O_2_ decomposition and CO_2_ generation.^27,28^ Consequently, the overall performance of these catalysts is not as effective as that of the directly synthesized Fe-ZSM-5.^27,28^ The key to improving the efficiency of this catalytic system is to maximize the amount of active Fe species while avoiding the production of unfavorable Fe species.

Here, we report that this aim can be achieved by an organic-template-free synthetic strategy. We found that whether or not TPA (the organic template) is used in the synthesis of Fe-ZSM-5 has a significant effect on the amount and configuration of Fe within the zeolite framework. In comparison with conventional templated Fe-ZSM-5 (denoted as Fe-Z5-C), Fe-ZSM-5 synthesized without using TPA (denoted as Fe-Z5-TF) has more framework Fe atoms, featuring lower coordination symmetry, higher dispersion, and a stronger tendency to migrate out of the zeolite framework. Therefore, significantly more extra-framework Fe species can be generated in Fe-Z5-TF than in Fe-Z5-C during the calcination process used to convert the catalyst into its H-form. Furthermore, unlike Fe-Z5-C, which produces multiple extra-framework Fe species (mainly binuclear Fe), Fe-Z5-TF produces almost exclusively mononuclear Fe species in the form of [(H_2_O)_2_–Fe(III)–OH]^2+^, which are highly active for CH_4_ oxidation. These factors together lead to a significant increase in the number of active Fe sites in Fe-ZSM-5 prepared via organic-template-free synthesis. Consequently, when used in the CH_4_ oxidation reaction with 0.5 M H_2_O_2_ in water at 75 °C, the H-form of Fe-Z5-TF (Fe-HZ5-TF) exhibited a high yield of C_1_ oxygenates (109.4 mmol g_cat_^–1^ h^–1^) with a HCOOH selectivity of 91.1%. This activity is more than 5 times higher than the value previously reported for Fe-ZSM-5 under similar conditions (19.9 mmol g_cat_^–1^ h^–1^).^34^ We also investigated CH_4_ activation at mononuclear Fe sites and the subsequent conversion to C_1_ oxygenates. The results suggest that H_2_O_2_ oxidizes mononuclear Fe to generate [(H_2_O)_3_–Fe(IV)=O)]^2+^, which in turn acts as a catalytically active center for the homolytic cleavage of the C–H bond in CH_4_ and radical-driven sequential oxidative conversions.

## Results and Discussion

### Preparation of the Fe-ZSM-5 Catalysts

The active sites in Fe-ZSM-5 are positively charged extra-framework Fe-oxo species bound to negatively charged framework Al sites, while the initial Fe content in the zeolite framework determines the maximum amount of extra-framework Fe produced via calcination. These two facts suggest that increasing the amount of framework Al and Fe in the initial Fe-ZSM-5 would be beneficial for generating more extra-framework Fe in the final calcined H-form catalyst. Compared to the conventional synthesis of ZSM-5 using TPA^+^, our proposed template-free synthesis allows more Al and Fe to be incorporated into ZSM-5 because the consequent high-density negative charges on the framework can be balanced easily by the smaller inorganic cations (Na^+^) in the synthetic system.^35−37^ Moreover, Fe-ZSM-5 synthesized using the template-free method can generate a higher proportion of desired extra-framework Fe species during calcination compared to conventional Fe-ZSM-5.

Under the synthetic conditions employed in this study, the Si/Al ratio of Fe-ZSM-5 synthesized with TPA^+^ was >25,^38,39^ whereas the Si/Al ratio of Fe-ZSM-5 synthesized without TPA^+^ (i.e., the template-free approach) was in the range of 12–20.^40^ For both systems, the maximum achievable amount of Fe in the zeolite framework is ∼10 mol % that of the Al (i.e., Al/Fe ≈ 10). As observed using ultraviolet–visible (UV–vis) spectroscopy (Figure S1), attempts to introduce more Fe to the zeolite by adding more Fe precursors resulted in the generation of iron oxide clusters or particles.

**Figure 1:**
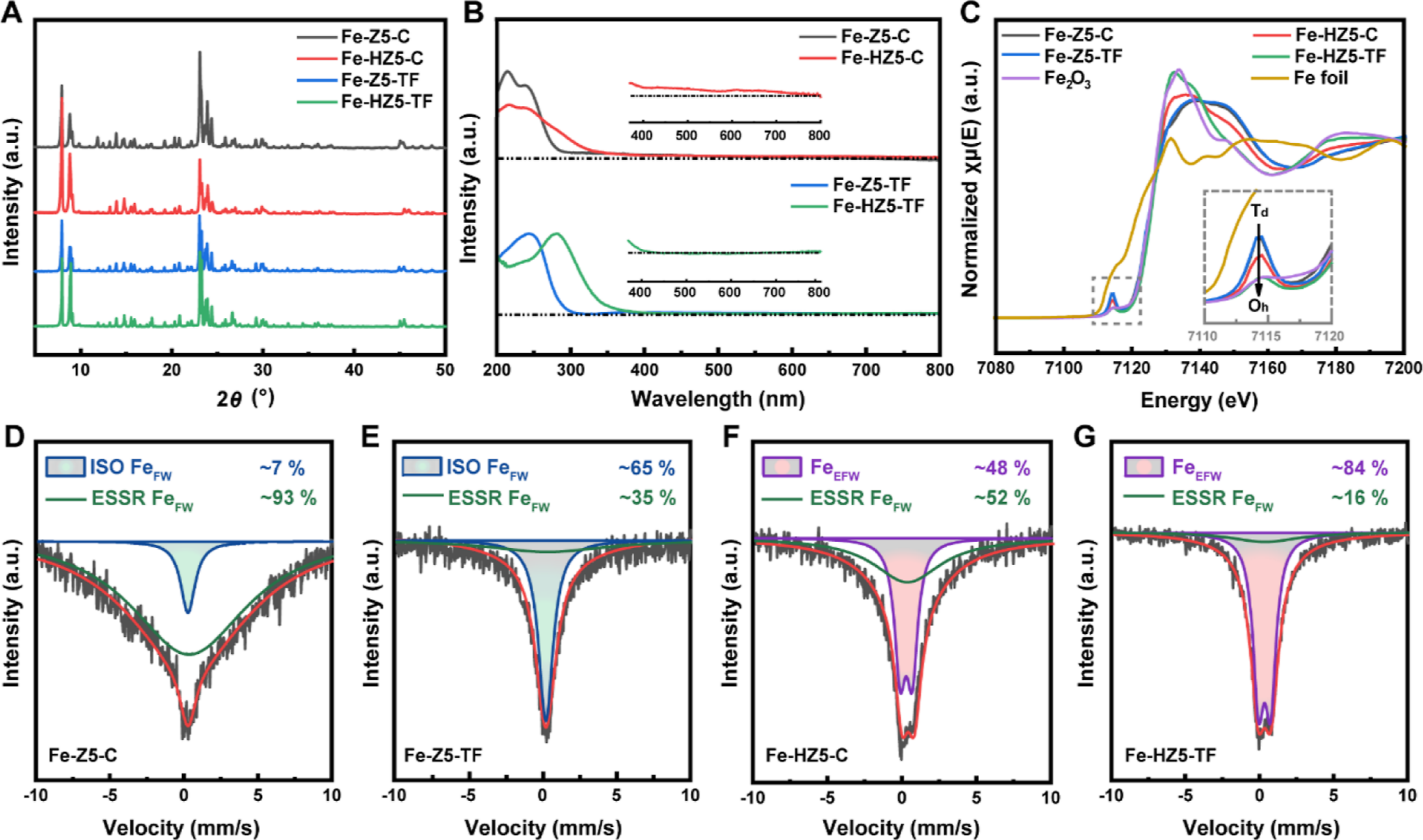
Characterization of Fe-Z5-C, Fe-HZ5-C, Fe-Z5-TF, and Fe-HZ5-TF. (A) PXRD patterns. (B) DR UV–vis spectra. The inset shows the magnified spectra of Fe-HZ5-C and Fe-HZ5-TF from 370 to 800 nm. (C) Fe K-edge XANES spectra. The inset shows the magnified pre-edge region of the spectra. (D–G) ^57^Fe Mössbauer spectra for the four catalysts with the Fe species and their proportions indicated. ISO Fe_FW_, ESSR Fe_FW_, and Fe_EFW_ refer to isolated framework Fe, framework Fe with the enhanced spin–spin relaxation effect, and extra-framework Fe species, respectively.

Two representative Fe-ZSM-5 samples were selected for detailed analysis: one synthesized via the conventional method with TPA^+^ (Si/Al = 33.5; Al/Fe = 10.8) and the other via the template-free method (Si/Al = 17.9; Al/Fe = 12.5) (Fe-Z5-C and Fe-Z5-TF, respectively). Before catalytic testing for CH_4_ oxidation, the as-synthesized samples underwent ion exchange with ammonium followed by calcination to convert them to H-form zeolites (Fe-HZ5-C and Fe-HZ5-TF, respectively). Table 1 lists the chemical composition of these four samples. Powder X-ray diffraction (XRD) revealed that the four samples were pure phases with an MFI structure (Figure 1A). No XRD peaks associated with iron oxides were observed, even for the H-forms, indicating that Fe was highly dispersed in the zeolite framework or micropores without forming bulk crystalline phases.

**Table 1:** Properties of Various Fe-ZSM-5 Catalysts

				Fe species (%)^b^					
samples	Fe loading (wt %)^a^	Si/Al^a^	Al/Fe^a^	ISO Fe_FW_	ESSR Fe_FW_	Fe_EFW_	extra-framework Fe (wt %)	Al pairs (%)^c^	Al pairs/Fe_2_ (or Fe_1_)^d^ (molar ratio)
Fe-Z5-C	0.24	34.5	11.1	∼7	∼93	0	0	9.8	
Fe-HZ5-C	0.25	33.5	10.8	0	∼52	∼48	0.12	9.8	1.25
Fe-Z5-TF	0.41	17.1	12.5	∼65	∼35	0	0	28.8	
Fe-HZ5-TF	0.39	17.9	12.5	0	∼16	∼84	0.33	28.8	1.78

a Determined using ICP-OES.

b Determined using ^57^Fe Mössbauer spectroscopy; ISO Fe_FW_, ESSR Fe_FW_, and Fe_EFW_ refer to isolated framework Fe, framework Fe with the enhanced spin–spin relaxation effect, and extra-framework Fe species, respectively.

c Proportion of Al pairs in total Al species derived from the ICP-OES results for Co-exchanged zeolites.

d Fe_2_ and Fe_1_ represent binuclear Fe and mononuclear Fe, respectively.

### Identification of Active Fe Species in the Fe-ZSM-5 Catalysts

Diffuse reflectance UV–vis spectroscopy is frequently used to analyze Fe species in zeolites because the absorption band associated with ligand-to-metal charge transfer (CT) is sensitive to the coordination geometry and environment, with framework Fe having adsorption bands in the range of 200–250 nm, compared to 250–350 nm for isolated and oligomeric extra-framework Fe confined in zeolite channels, 350–450 nm for larger Fe clusters, and >450 nm for bulk iron oxide particles. Consistent with the literature, Fe-Z5-C exhibited two discernible CT bands in the UV–vis spectrum (Figure 1B), centered at 211 and 245 nm, which are characteristic of isomorphously incorporated, tetrahedrally coordinated Fe^3+^ ions within the zeolite framework, corresponding to *t*_1_ → *t*_2_ and *t*_1_ → *e* transitions, respectively.^41,42^ In contrast, Fe-Z5-TF showed only one intense CT band at 248 nm (Figure 1B), suggesting an unusual framework Fe coordination geometry or environment. In previous research on template-free synthesized Fe-ZSM-5,^43,44^ the same single-band absorption was observed and assigned to the framework Fe without questioning why it differed from the typical double-band absorption. In another study, the double-band and single-band absorption was attributed to Fe^3+^ in a perfect and distorted tetrahedral (*T*_d_) coordination environment, respectively.^33^

Compared to Fe-Z5-C, Fe-HZ5-C exhibited a significantly broader spectrum, in which the two bands at 211 and 245 nm remained discernible but with reduced intensity, and the long tail covered a broad range up to >500 nm (Figure 1B). These observations are consistent with results reported elsewhere,^22,27−29^ indicating that the partial migration of Fe atoms yielded various extra-framework Fe species, mainly oligomeric species and trace amounts of clusters and particles. Interestingly, the sample prepared using the template-free method in the present study exhibited distinctive behavior. The UV–vis spectrum of Fe-HZ5-TF was similar in shape to Fe-Z5-TF but shifted toward higher wavelengths by ∼30 nm, with no absorption at >400 nm (Figure 1B), which suggests that almost all Fe migrates out of the zeolite framework during calcination to form homogeneous extra-framework Fe species confined in the micropores.

In the X-ray absorption near-edge structure (XANES) spectra for Fe-Z5-TF and Fe-Z5-C (Figure 1C), there was a pre-edge peak at 7114 eV, which is indicative of Fe^3+^ in the tetrahedral zeolite framework, corresponding to the transition from 1s to 3d-like levels (i.e., the strong mixing of 3d and 4p metal orbitals due to electric-quadrupole coupling).^45,46^ This peak was almost absent for Fe^3+^ with centrosymmetric octahedral coordination (e.g., in Fe_2_O_3_, which was used as a reference). Compared to Fe-Z5-C, the pre-edge peak of Fe-HZ5-C was less intense but still clearly visible, which corresponds to the partial transformation of framework Fe to octahedrally coordinated extra-framework Fe. In the XANES spectrum of Fe-HZ5-TF, the pre-edge peak intensity of Fe-HZ5-TF was very low, indicating the complete migration of framework Fe. Therefore, the conclusions drawn from XANES were consistent with those from the UV–vis analysis.

^57^Fe Mössbauer spectroscopy was conducted to quantitatively analyze Fe species in different states. The spectra for Fe-Z5-C and Fe-Z5-TF were deconvoluted into a singlet component with an isomer shift (IS) of ∼0.25 mm/s and a broad magnetic relaxation component with an IS of ∼0.36 mm/s (Figure 1D,E), both of which were related to tetrahedrally coordinated high-spin Fe^3+^ in the zeolite framework.^47^ The singlet component corresponded to isolated framework Fe atoms that were far apart from each other (denoted as ISO Fe_FW_), while the broad magnetic relaxation component corresponded to framework Fe atoms that were close enough to exhibit the enhanced spin–spin relaxation effect (denoted as ESSR Fe_FW_).^48,49^ Quantitative analysis revealed that most of the Fe (∼93%) in Fe-Z5-C was ESSR Fe_FW_, whereas most of the Fe (∼65%) in Fe-Z5-TF was ISO Fe_FW_ (Table 1), meaning that Fe-Z5-TF had a higher dispersion degree of framework Fe than Fe-Z5-C. The high proportion of ESSR Fe_FW_ in Fe-Z5-C can be attributed to the preferential localization of TPA^+^ at the channel intersections in ZSM-5, resulting in the enrichment of trivalent Fe therein.^50^ In contrast, the distribution of Fe in Fe-Z5-TF is not restrained by the organic template and thus is more dispersed and homogeneous.

In the ^57^Fe Mössbauer spectra for Fe-HZ5-C and Fe-HZ5-TF, a doublet component (IS = 0.32 mm/s and quadrupole splitting = 0.81 mm/s) was observed (Figure 1F,G), which corresponded to extra-framework octahedral Fe^3+^ species (denoted as Fe_EFW_). The spectra could be deconvoluted with satisfactory accuracy based on the coexistence of Fe_EFW_ and ESSR Fe_FW_ (Figure 1F,G). The results indicated that during the transformation of Fe-Z5-C to Fe-HZ5-C, ∼48% of the framework Fe was converted to extra-framework Fe; in comparison, this value was as high as ∼84% for the transformation of Fe-Z5-TF to Fe-HZ5-TF (Table 1).

Collectively, these spectroscopy characterization results identified two significant differences between the Fe-ZSM-5 samples synthesized with and without the organic template. First, the framework Fe in Fe-Z5-C and Fe-Z5-TF exhibited different coordination geometries (i.e., *T*_d_ vs distorted *T*_d_) and distribution (i.e., ESSR Fe_FW_ vs ISO Fe_FW_ as the primary species). Second, compared to Fe-Z5-C, a significantly higher proportion of Fe in Fe-Z5-TF migrates out of the zeolite framework to form extra-framework Fe species when converted to the H-form. It is reasonable to speculate that the first observation was the cause of the second. Compared to Fe-Z5-C, Fe-Z5-TF had an inherently higher Fe content and higher Fe migration, and consequently, it yielded approximately 3 times more extra-framework Fe in the H-form catalyst (Table 1).

**Figure 2:**
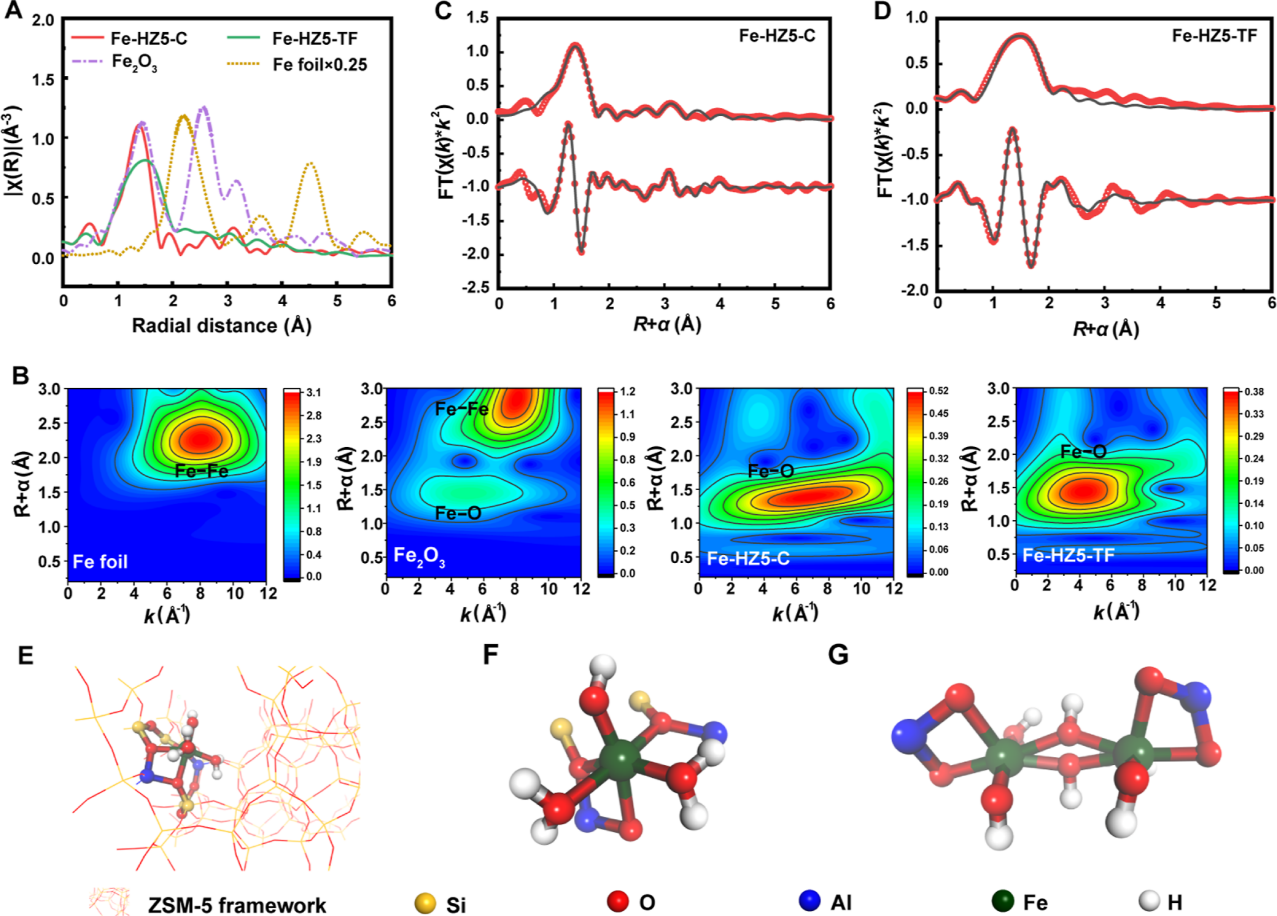
Determination of extra-framework Fe species. (A,B) FT-EXAFS spectra (A) and WT-EXAFS contour plots (B) for Fe-HZ5-C and Fe-HZ5-TF in comparison with Fe foil and Fe_2_O_3_, which were used as reference materials. (C,D) Fe K-edge EXAFS (points) and fitting curve (line) for Fe-HZ5-C (C) and Fe-HZ5-TF (D) shown in *R*-space. In each panel, the upper and lower lines are the FT magnitude and imaginary components, respectively. (E) DFT-optimized structural model of mononuclear Fe bound to an Al pair at a β site in the ZSM-5 framework. (F) The same mononuclear Fe structural model as in (E), with the zeolite framework removed for clarity. (G) Structural model of binuclear Fe species reported in the literature.

Figure 2A presents the *k*^2^-weighted Fourier-transformed extended X-ray absorption fine structure (EXAFS) spectra for Fe-HZ5-C and Fe-HZ5-TF using the Fe foil and Fe_2_O_3_ as references. Unlike the reference materials, which have multiple peaks associated with Fe–Fe interactions, Fe-HZ5-C and Fe-HZ5-TF exhibited only one intense peak at 1.4 Å (uncorrected for the phase shift) corresponding to Fe–O scattering, suggesting that Fe was dispersed atomically without the formation of bulk oxides. The EXAFS spectra were analyzed further using wavelet transforms (WTs) based on Morlet wavelets with an optimal resolution (Figure 2B). A comparison of the WT contour plots for the Fe foil and Fe_2_O_3_ revealed that the intensity maxima at ∼4.0 and ∼8.0 Å^–1^ correspond to Fe–O and Fe–Fe paths, respectively (Figure 2B). The WT contour plot for Fe-HZ5-TF exhibited no sign of the Fe–Fe path but only a concentrated intensity distribution with a maximum at 4.0 Å^–1^, which corresponds to the Fe–O path according to the reference materials. The absence of a Fe–Fe path suggests that the extra-framework Fe species in Fe-HZ5-TF are predominantly mononuclear Fe. In contrast, Fe-HZ5-C had a diffuse intensity distribution along the *k*-vector direction, centered at 7.0 Å^–1^. Considering that its *R* + α value (∼1.4 Å) is in the range of the Fe–O bond distance, this diffuse intensity can be attributed to a Fe–O path perturbed by a coexisting Fe–Fe path, suggesting that Fe-HZ5-C contains multinuclear extra-framework Fe species. One possible reason why Fe-HZ5-C does not show a separate Fe–Fe path as observed in Fe_2_O_3_ (Figure 2B) is the low content and small size of the multinuclear extra-framework Fe clusters.

Least-squares EXAFS fitting was employed to obtain the average fine structure parameters, including the interatomic distance (ID), coordination number (CN), and Debye–Waller factor (σ^2^) (Figure 2C,D and Table 2). For Fe-HZ5-C, given the presence of framework Fe and extra-framework multinuclear Fe, three paths (Fe–O, Fe–Al, and Fe–Fe) were included simultaneously for the fitting. The results revealed a dominant Fe–O path (ID: 1.90 Å) with a CN of 4.6, a Fe–Al path (ID: 2.79 Å) with a CN of 0.8, and a Fe–Fe path (ID: 3.04 Å) with a CN of 0.7 (Figure 2C and Table 2). Given that the amount of framework tetrahedral Fe and extra-framework Fe in Fe-HZ5-C was very similar (52:48), the average Fe–O CN (≈5) confirmed that the extra-framework Fe species were octahedrally coordinated, while the average Fe–Fe CN (≈0.5) indicated that the extra-framework Fe species were predominantly binuclear Fe (because binuclear Fe has a theoretical Fe–Fe CN of 1).

**Table 2:** Fe K-Edge EXAFS Curve Fitting Parameters for Various Fe-ZSM-5 Samples^a^

samples	paths	*R* (Å)	CN	δ^2^ (×100 Å^2^)	Δ*E*_0_ (eV)	*R*-factor
Fe-Z5-C	Fe–O	1.85 (0.02)	4.0 (0.8)	0.3 (0.2)	4 (3)	0.015
Fe-HZ5-C	Fe–O	1.90 (0.03)	4.6 (0.5)	0.3 (0.1)	5 (1)	0.012
	Fe–Al	2.79 (0.05)	0.8^b^	1.2 (0.3)		
	Fe–Fe	3.04^a^	0.7 (0.4)	0.9 (0.2)		
Fe-Z5-TF	Fe–O	1.84 (0.01)	4.1 (0.7)	0.4 (0.1)	5 (2)	0.009
Fe-HZ5-TF	Fe–O1	1.86 (0.09)	1.9^c^	0.3^b^	2 (7)	0.011
	Fe–O2	2.03 (0.08)	4.1 (1.4)	0.3^b^		
Fe-HZ5-TF	Fe–O1	1.89 (0.04)	2.0^b^	0.7 (0.5)	17 (8)	0.024
	Fe–O2	2.08 (0.05)	4.0^d^	0.8 (0.5)		
	Fe–Al	2.76 (0.12)	1.7 (1.1)	0.5 (0.2)		
	Fe–Fe	2.95 (0.10)	0.3^b^	0.5 (0.6)		

a *R*, distance between absorber and backscatter atoms; CN, coordination number; δ^2^, Debye-Waller factor to account for thermal and structural disorder; Δ*E*_0_, inner potential correction; *R*-factor indicates the goodness of the fit.

b Restrained value.

c Set value.

d Define: CN(Fe–O2) = 6-CN(Fe–O1).

Because Fe-HZ5-TF had only mononuclear extra-framework Fe and a small amount of framework Fe, the fitting of its EXAFS spectrum did not involve Fe–Al and Fe–Fe paths. Compared to Fe-HZ5-C, the raw spectrum for Fe-HZ5-TF had a markedly broader Fe–O peak (Figure 2A), suggesting that there may be multiple Fe–O paths in the first shell of the Fe center. The best-fitting result (*R*-factor: 0.011) confirmed the presence of two independent Fe–O paths, one with an ID of 1.86 Å and a CN of 1.9 and the other with an ID of 2.03 Å and a CN of 4.1 (Figure 2D and Table 2). The total CN of ∼6 was consistent with the conclusion that most of the Fe in Fe-HZ5-TF was extra-framework Fe with octahedral coordination. The attempt to include a Fe–Fe path in the EXAFS fitting for Fe-HZ5-TF resulted in a higher *R*-factor of 0.024 (Figure S2 and Table 2). The resulting average CN for Fe–Fe was as low as 0.3, further confirming that Fe-HZ5-TF contained only trace amounts of multinuclear Fe species if any.

These characterization results raise the question of why extra-framework Fe takes a binuclear form in Fe-HZ5-C but a mononuclear form in Fe-HZ5-TF. This difference can be explained by the higher Al content in Fe-HZ5-TF than in Fe-HZ5-C because more Al means more Al pairs and thus more anchoring sites to promote the dispersion of extra-framework Fe in a more isolated form. This speculation was verified using an established method to measure the number of Al pairs. Two Fe-free ZSM-5 samples were prepared using TPA and the template-free method (denoted as Z5-C and Z5-TF, respectively) for this to avoid interference from Fe. In brief, Co^2+^ was introduced into the calcined zeolites via ion exchange. Due to the one-to-one correspondence between Co^2+^ and Al pairs, the number of Al pairs can be estimated from the Co uptake measured using inductively coupled plasma mass spectrometry,^51,52^ while the distribution of Al pairs in the zeolite framework can be inferred from the UV–vis spectra for dehydrated Co-exchanged zeolites (Figure S3). The results showed that 28.8% of the total Al in Z5-TF forms Al pairs, compared to only 9.8% for Z5-C, which is consistent with expectations. In both samples, Al pairs are predominantly located at the intersections of the straight and sinusoidal channels (i.e., the β sites). The data summarized in Table 1 and Figure S4 revealed two interesting facts. First, the absolute number of Al pairs in Z5-TF was approximately 5 times higher than in Z5-C, which perfectly matches the number ratio of mononuclear Fe in Fe-HZ5-TF to binuclear Fe in Fe-HZ5-C. Second, the numbers of Al pairs are sufficient to anchor the corresponding active Fe centers in both systems.

Based on the CN and primary locations determined from the above experimental results, three models for the octahedral mononuclear [(H_2_O)_*n*=1,2,3_–Fe(III)–OH]^2+^ complex anchored by an Al pair at the β site were constructed and optimized by the DFT method (Figure S5A–C). Of the three optimized models, [(H_2_O)_2_–Fe(III)–OH]^2+^ bound to three zeolite framework oxygen (O_f_) atoms (Figure 2E and Table S1) exhibited the closest agreement with the EXAFS fitting results in terms of the Fe–O bond lengths (Table 2). The possibility of stabilizing mononuclear Fe with a single Al site rather than an Al pair was also investigated using [(H_2_O)_2_–Fe(III)–(OH)_2_]^+^ complexes with different ligand configurations at the octahedral apical sites (Figure S5). It was found that the Fe–O bond lengths obtained from the most energetically stable configurations (Figure S5F and Table S1) did not conform to the EXAFS results (Table 2). Therefore, it was concluded that the mononuclear Fe species in Fe-HZ5-TF was in the form of [(H_2_O)_2_–Fe(III)–OH]^2+^ bound to three O_f_ atoms (Figure 2F). Figure 2G displays the binuclear Fe model previously described in the literature for conventional Fe-ZSM-5 as a comparison.^26,28^

### Catalytic Performance

The prepared Fe-HZ5-C and Fe-HZ5-TF catalysts were evaluated for the selective oxidation of CH_4_. The reactions were conducted in a 100 mL autoclave reactor containing 10 mL of an aqueous H_2_O_2_ solution (0.5 M) and 27 mg of the catalyst at 75 °C and 30.5 bar CH_4_ for 25 min. All tested catalysts exhibited a similar selectivity, with HCOOH as the predominant product and other minor products including CH_3_OH, CO_2_, CH_3_OOH, and HOCH_2_OOH. Therefore, we focus the following discussion on catalyst activity and use the overall C_1_ oxygenate productivity (μmol) as the main criterion for comparison.

**Figure 3:**
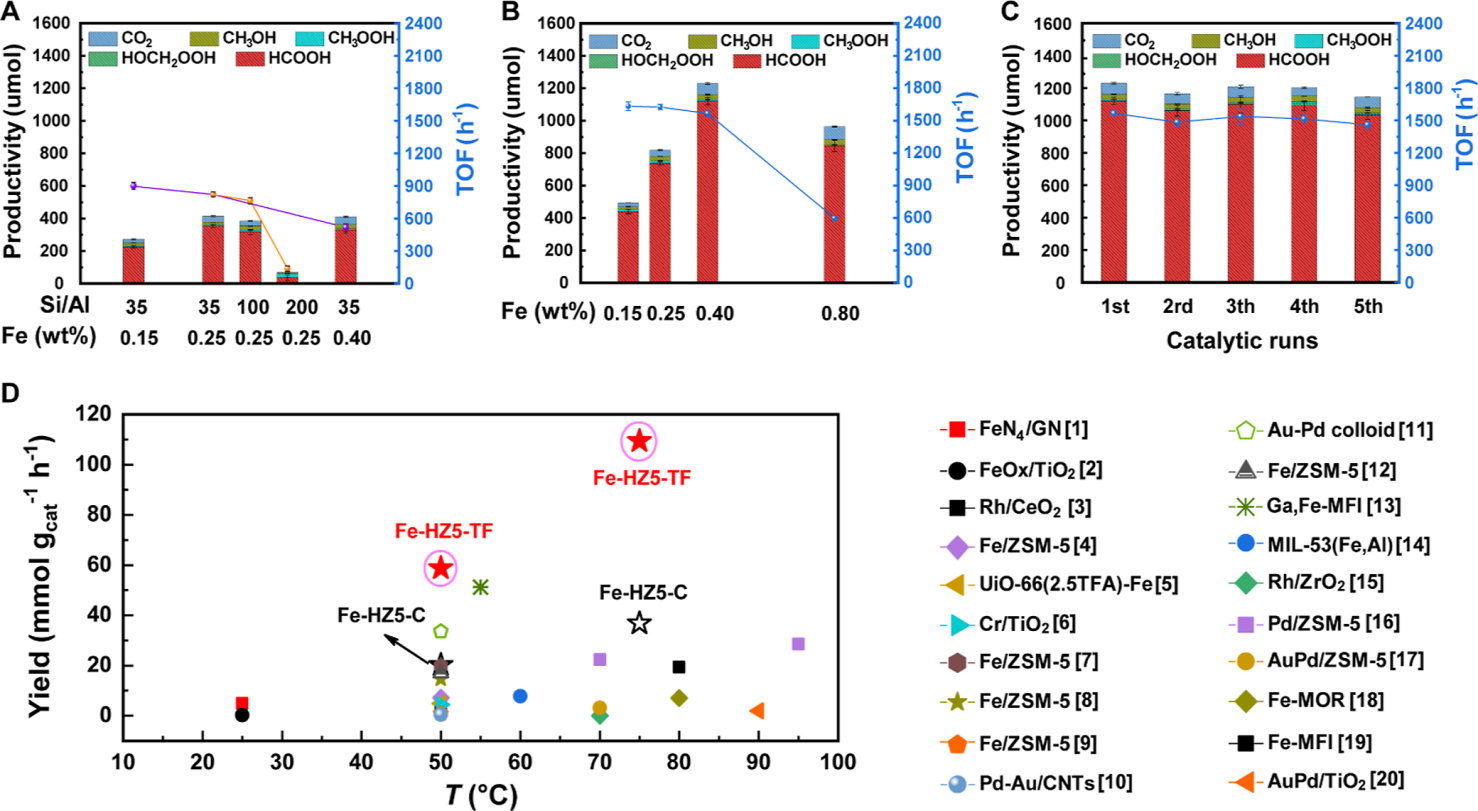
Catalytic performance of various catalysts in the selective oxidation of methane. (A) Fe-HZ5-C with different Si/Al ratios and Fe loadings. (B) Fe-HZ5-TF with different Fe loadings. (C) Results of Fe-HZ5-TF (Fe loading: 0.40 wt %) for five successive reaction runs. Reaction conditions for (A–C): 27 mg of catalyst, 10.0 mL of H_2_O_2_ aqueous solution (0.5 M), 30.5 bar CH_4_, 75 °C, and 25 min. The error bars represent the standard deviations from three parallel measurements. (D) Performance comparison of Fe-HZ5-TF with state-of-the-art catalysts. Numbers in square brackets correspond to the entry numbers in Table S2.

We first tested a series of Fe-HZ5-C samples with a fixed Fe content (0.25 wt %) but different Si/Al ratios (approximately 200, 100, and 35). The sample with the highest Al content (i.e., Si/Al ≈ 35) exhibited the highest activity (Figure 3A), which can be attributed to the favorable migration of framework Fe and the dispersion of extra-framework Fe associated with high Al levels.^27^ Therefore, the Si/Al ratio was fixed at 35 (i.e., close to the highest Al content available for Fe-Z5-C) in the subsequent tests to examine the effect of Fe loading on the activity of Fe-HZ5-C. To this end, two additional Fe-HZ5-C samples (Si/Al ≈ 35) were prepared, which contained less and more Fe (0.15 and 0.40 wt %, respectively) compared with the standard Fe-HZ5-C sample (0.25 wt %). As shown in Figure 3A, both samples produced lower C_1_ oxygenate levels than the standard Fe-HZ5-C. In particular, the C_1_ productivity of Fe-HZ5-C with 0.15, 0.25, and 0.40 wt % Fe was 271.8, 413.9, and 410.9 μmol, corresponding to a turnover frequency (TOF) of 899.6, 821.9, and 518.8 h^–1^, respectively. These results indicated that at low Fe loads, the productivity increased almost in proportion with the Fe content, whereas there was an optimal Fe loading beyond which further increases in Fe led to a decrease in productivity. More systematic analysis revealed that the optimal Fe loading was dependent primarily on the Al content in the zeolite framework (i.e., Al/Fe ≈ 10). This can be attributed to the fact that active (i.e., binuclear) Fe species require Al pairs to stabilize them, while excess Fe relative to framework Al leads to the formation of clusters and particles that adversely affect the reaction through the nonselective decomposition of H_2_O_2_.^27,28^ UV–vis spectra confirmed that the Fe-HZ5-C sample with 0.4 wt % Fe had more pronounced absorption at >350 nm compared to the samples with lower Fe content (Figure S6).

Based on these analyses, the optimal composition of Fe-HZ5-C was determined to be Si/Al = 33.5 and Al/Fe = 10.8 (i.e., a Fe loading of 0.25 wt %). Under typical reaction conditions, the optimized Fe-HZ5-C exhibited the highest achievable yield at 36.8 mmol of C_1_ products per gram of the catalyst per hour (mmol g_cat_^–1^ h^–1^) (Figure 3D). When the reaction temperature was reduced to 50 °C, the optimized Fe-HZ5-C had a C_1_ oxygenate yield of 20.2 mmol g_cat_^–1^ h^–1^ (Figure 3D), which was similar to the previously reported highest value for conventional Fe-ZSM-5 under similar conditions (Table S2).

Because the Si/Al ratio of Fe-HZ5-TF cannot vary greatly, we only investigated the effect of the Fe loading on its activity at a fixed Si/Al ratio of ∼18. When the Fe loading was low (0.15–0.40 wt %), the oxygenate productivity was approximately proportional to the Fe content, while the TOF was almost constant (Figure 3B). Increasing the Fe loading to 0.80 wt % resulted in lower oxygenate productivity and a significant drop in the TOF (Figure 3B). These observations were consistent with the Fe-HZ5-C system and were explained and verified in a similar manner (Figure S7). On the other hand, the observed catalytic activity for the optimal Fe-HZ5-TF (0.40 wt % Fe) was approximately 3 times higher than that for the optimal Fe-HZ5-C (0.25 wt % Fe). Specifically, Fe-HZ5-TF (0.40 wt % Fe) produced 1230.4 μmol of oxygenates (Figure 3B), corresponding to 109.4 mmol g_cat_^–1^ h^–1^ (Figure 3D). When the reaction temperature was lowered to 50 °C, the oxygenate productivity was 667.0 μmol (59.3 mmol g_cat_^–1^ h^–1^), which was approximately 3 times higher than that of Fe-HZ5-C under the same conditions (Figure 3D). Given that Fe-HZ5-TF (0.40 wt % Fe) had approximately 3 times more active Fe species than Fe-HZ5-C (0.25 wt % Fe) (Table 1), the TOF based on active Fe species was similar for both catalysts (Figure S8).

There are small but discernible differences between Fe-HZ5-TF and Fe-HZ5-C in product selectivity. Specifically, Fe-HZ5-TF (0.40 wt % Fe) shows slightly higher formic acid selectivity (91.1% vs 85.6%) and lower CO_2_ selectivity (5.7% vs 8.6%) than Fe-HZ5-C (0.25 wt % Fe), which suggests that compared with binuclear Fe, mononuclear Fe can inhibit the overoxidation of HCOOH to CO_2_ to a certain extent. To verify this conjecture, we performed the reaction using HCOOH as the reactant under the typical conditions used in this study. The results revealed that, as expected, Fe-HZ5-TF produced less CO_2_ than Fe-HZ5-C, despite its much higher extra-framework Fe content (Figure S9).

Figure 3D compares the oxygenate yields obtained from various catalysts at different reaction temperatures and similar H_2_O_2_/catalyst weight ratios, showing that Fe-HZ5-TF has the highest activity of the reported catalysts (see Table S2 for more detail). The high conversion capacity per unit mass of Fe-HZ5-TF can be attributed to the high number of active Fe sites, which were maximized using the template-free synthesis method. Although the Fe loading on ZSM-5 can be easily increased to higher levels using postsynthesis methods, such as wet impregnation, ion exchange, and solid-state ion exchange, the Fe-ZSM-5 catalysts prepared in this way contained many undesirable clusters and particles and exhibited lower activity than Fe-HZ5-TF (Figure S10). In addition to the high activity, Fe-HZ5-TF demonstrated good recyclability without a noticeable drop in productivity over five consecutive reaction runs (Figure 3C), indicating that the extra-framework mononuclear Fe species were firmly confined to the zeolite channels. Inductively coupled plasma–optical emission spectrometry (ICP-OES) analysis of the postreaction solution indicated that no iron was leached into the solution during the reactions, which is consistent with the observed excellent catalyst reusability. Furthermore, our preliminary results showed that Fe-HZ5-TF can also be used for the selective oxidation of higher alkanes, such as ethane and propane, to produce higher-value oxygenates (Tables S3 and S4).

### Methane Conversion at Mononuclear Fe Sites

Conventional Fe-ZSM-5 is generally considered to activate CH_4_ molecules through a synergistic effect at binuclear Fe sites. Because mononuclear Fe can also act as an active site for CH_4_ oxidation in the presence of H_2_O_2_, as demonstrated in this study, it is crucial to understand how CH_4_ is activated and converted by mononuclear Fe.

**Figure 4:**
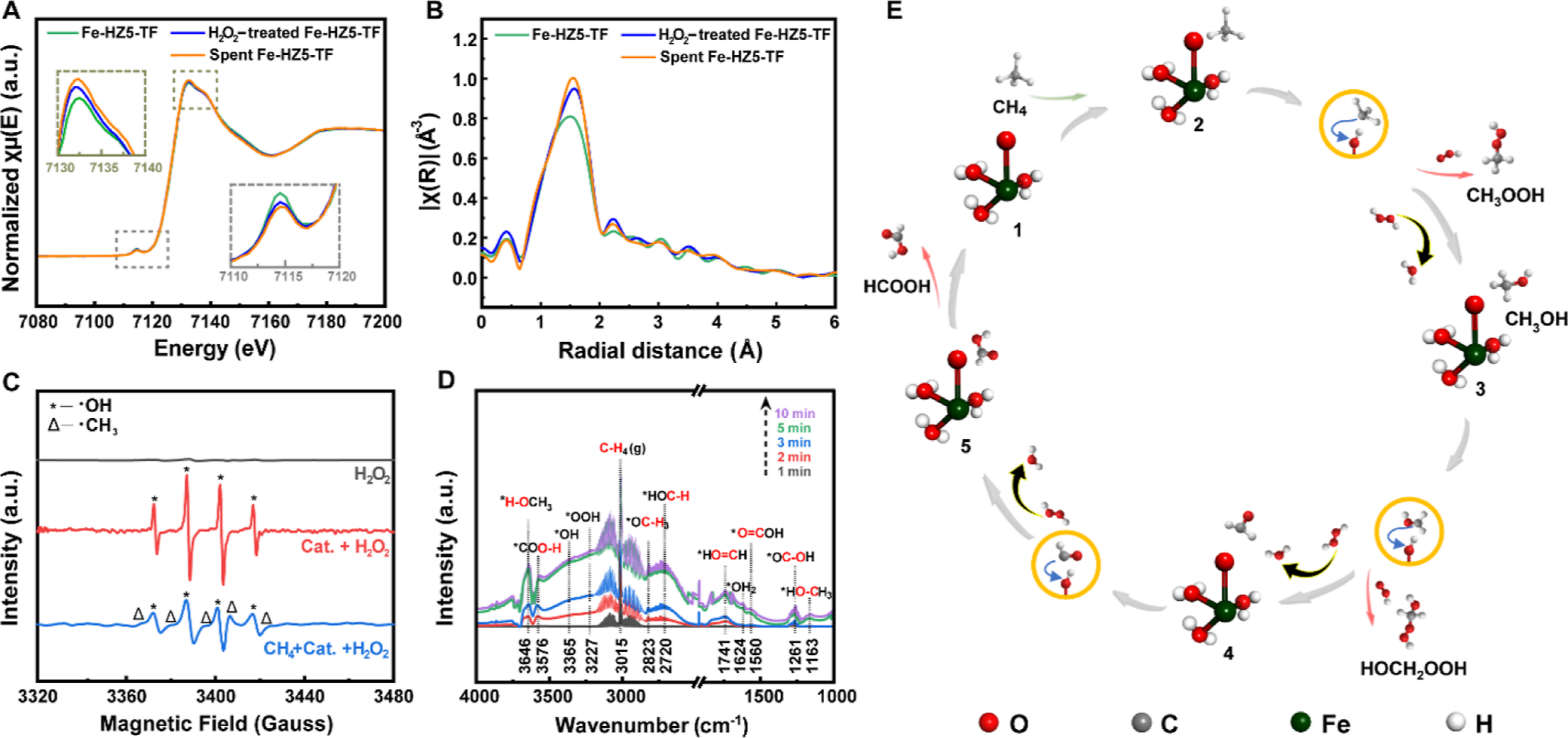
Investigation of the reaction pathway and mechanisms. (A) Fe K-edge XANES spectra of pristine, H_2_O_2_-treated, and spent Fe-HZ5-TF catalysts. The insets are magnified spectra of the pre-edge and white-line regions. (B) FT-EXAFS spectra of pristine, H_2_O_2_-treated, and spent Fe-HZ5-TF catalysts. (C) EPR spectra of the reaction solution in different states: H_2_O_2_ solution alone, H_2_O_2_ solution with the catalyst (Fe-HZ5-TF), and H_2_O_2_ solution with Fe-HZ5-TF and CH_4_ involved. Reaction conditions: 4.0 mL of H_2_O, 50 mg of DMPO, 0.5 mL of H_2_O_2_ solution (30%), 27 mg of the catalyst (if applied), and 30.5 bar of CH_4_ (if applied). DMPO was used as the radical trapping agent. The reactions were terminated at 2 min. (D) In situ time-resolved DRIFTS spectra acquired for Fe-HZ5-TF at 75 °C under a gas flow containing CH_4_ and H_2_O_2_. (E) Schematic illustration of the proposed radical-driven pathway for the selective oxidation of methane on mononuclear Fe active sites. The yellow circles highlight the generation of Fe–OH from high-valence Fe=O by abstracting H from the reaction intermediates.

To gain insights into this process, we characterized two samples using powder XRD, UV–vis, and X-ray absorption spectra: Fe-HZ5-TF treated with 0.5 M H_2_O_2_ (H_2_O_2_-treated Fe-HZ5-TF) and Fe-HZ5-TF recovered after the CH_4_ oxidation reaction (spent Fe-HZ5-TF). The results indicated that, following the addition of H_2_O_2_ or during the catalytic reaction, the mononuclear Fe species remained isolated and did not agglomerate to form Fe_*x*_O_*y*_ clusters or particles (Figure S11). However, compared to pristine Fe-HZ5-TF, the H_2_O_2_-treated and spent Fe-HZ5-TF both exhibited higher white line intensities and lower pre-edge peak intensities in the XANES spectra (Figure 4A). These spectral changes suggested that both the valence state and CN of the Fe center increased during contact with H_2_O_2_. Furthermore, H_2_O_2_-treated and spent Fe-HZ5-TF both demonstrated enhanced peak amplitudes for the Fe–O shell compared to pristine Fe-HZ5-TF (Figure 4B), also suggesting that the CN of Fe increased (see the fitting results in Figure S12 and Table S5).

Electron paramagnetic resonance (EPR) spectroscopy showed that adding Fe-HZ5-TF could promote the generation of ^•^OH radicals in the H_2_O_2_ solution (Figure 4C). EPR spectroscopy also detected ^•^OH and ^•^CH_3_ radicals in the solution after the CH_4_ selective oxidation reaction (Figure 4C). In situ diffuse reflectance-infrared Fourier-transform spectroscopy (DRIFTS) was also conducted to identify the CH_4_ oxidation reaction intermediates for Fe-HZ5-TF at 75 °C. As the reaction proceeded, three peaks of increasing intensity appeared at 3365, 3227, and 1624 cm^–1^, which could be assigned to ^•^OH, ^•^OOH, and ^•^OH_2_ (likely the result of ^•^OH extracting ^•^H from CH_4_ or −Fe–OH), respectively (Figure 4D).^53,54^ Ex situ EPR spectroscopic analysis did not detect ^•^OOH and ^•^OH_2_ radicals, possibly because of their low concentrations and short lifetimes. Moreover, characteristic peaks for HCOH at 2720 and 1741 cm^–1^ were observed in DRIFTS (Figure 4D), indicating the presence of HCOH as an intermediate product during the reaction.

Overall, CH_4_ activation requires high-valence Fe, while the subsequent conversion of CH_4_ is based on radical reactions. This is consistent with a previous finding that Fe(IV)=O, which is surrounded by weak field ligands to have a high-spin state for Fe, is reactive for the abstraction of H from CH_4_.^26,55^ Accordingly, the following CH_4_ activation/conversion process at mononuclear Fe sites is proposed. First, the initial mononuclear Fe(III) sites are oxidized by H_2_O_2_, generating Fe(IV)=O groups and ^•^OH radicals
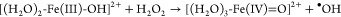


CH_4_ is then adsorbed onto the resulting Fe(IV)=O, followed by the homolytic cleavage of one of the C–H bonds to form Fe(III)–OH and ^•^CH_3_ radicals. These ^•^CH_3_ radicals subsequently react with ^•^OOH and ^•^OH radicals in solution to form CH_3_OOH and CH_3_OH. The resulting Fe(III)–OH can be reoxidized to Fe(IV)=O by H_2_O_2_ to restart this process (Figure 4E). The formation of other oxygenate products can be explained by the same C–H bond activation combined with radical reactions and subsequent deep oxidation (Figure 4E). This process does not require the simultaneous participation of two Fe centers in close proximity.

The evolution of various products was monitored during CH_4_ selective oxidation (Figure S13). The yields of CH_3_OOH and HOCH_2_OOH were the highest at the beginning of the reaction and then decreased monotonically over time. In contrast, the yields of HCOOH and CO_2_ increased continuously during the reaction, while the yield of CH_3_OH increased for the first 25 min of the reaction, but extending the reaction time further caused it to decrease. These results indicate the presence of peroxygenate-to-oxygenate conversion and sequential oxidation during the reaction.

We note that Yu et al. recently reported the preparation of mononuclear Fe species in zeolites and their role in catalyzing CH_4_ conversion.^25^ Our work differs from theirs mainly in two aspects. First, the wet impregnation method they used can only produce limited mononuclear Fe sites together with a considerable amount of undesired oligomeric clusters. They attempted to increase the mononuclear Fe content by reducing the Fe loading to 0.1 wt % but only obtained 66% mononuclear Fe. The absolute amount of mononuclear Fe obtained in their study is about one-fifth that of Fe-HZ5-TF, which explains the significant difference in C_1_ oxygenates productivity between the two systems under similar reaction conditions. Second, they concluded that methane interacts directly with individual Fe centers rather than oxo sites, whereas our conclusion is the opposite. In an in situ DRIFT experiment (Figure S14), we introduced two reactants (i.e., CH_4_ and H_2_O_2_) stepwise into an in situ cell containing Fe-HZ5-TF at 75 °C. Before switching to the second reactant, the cell was purged with He gas to remove the free molecules of the first reactant. When CH_4_ was introduced first, no oxygenate products were detected after the addition of H_2_O_2_ (Figure S14A). Interestingly, reversing the order of introduction of the two reactants (i.e., introducing H_2_O_2_ first) resulted in the production of formic acid, as revealed by the DRIFT spectra (Figure S14B). These results suggest that the activation of CH_4_ cannot occur directly at the Fe center but requires the generation of H_2_O_2_-derived reactive oxygen species at the Fe sites.

Oda et al. proposed a similar concept of maximizing the number of Fe sites in ZSM-5 to improve CH_4_ conversion efficiency.^56^ However, they employed a distinctly different method, synthesizing ZSM-5 exclusively with TPA as the template and introducing Fe through post-synthesis ion exchange. Their Fe-ZSM-5 catalyst achieved an oxygenate yield of >200 mmol g_cat_^−1^ h^−1^ at 50 °C, but with an unusually high H_2_O_2_/catalyst ratio.

## Conclusions

Organic-template-free synthesis can efficiently increase the number of active Fe sites in Fe-ZSM-5 by regulating the amount, dispersion, and coordination symmetry of the framework Fe. Moreover, unlike conventional Fe-ZSM-5, which has binuclear Fe as the main extra-framework Fe species, Fe-ZSM-5 prepared using organic-template-free synthesis (Fe-HZ5-TF) has almost exclusively mononuclear Fe species. Comprehensive and detailed spectroscopic characterization combined with DFT calculations showed that the mononuclear Fe was in the form of [(H_2_O)_2_–Fe(III)–OH]^2+^ grafted onto the ZSM-5 framework via interactions with Al pairs. Although mononuclear Fe has an intrinsic activity similar to that of binuclear Fe, the density (i.e., the number) of active Fe sites in Fe-HZ5-TF was approximately 3 times higher, leading to a corresponding 3-fold increase in catalytic efficiency, compared to the optimized conventional Fe-ZSM-5. When used for selective CH_4_ oxidation with 0.5 M H_2_O_2_ at 75 °C, Fe-HZ5-TF exhibited a C_1_ oxygenate yield of 109.4 mmol g_cat_^–1^ h^–1^, outperforming various state-of-the-art catalysts. During the reaction, [(H_2_O)_2_–Fe(III)–OH]^2+^ was first oxidized by H_2_O_2_ to [(H_2_O)_3_–(Fe(V)=O)]^2+^, which in turn acted as a catalytic center to activate CH_4_ and facilitate the subsequent sequential oxidative transformations, eventually producing HCOOH with high selectivity. In addition to its higher efficiency in terms of generating active Fe sites, organic-template-free synthesis is more cost-effective and environmentally friendly than conventional synthesis, making it highly desirable for the large-scale industrial production of catalysts.

## Supporting Information Available

Experimental and computational sections and details about DR UV–vis, EXAFS fitting, WT-EXAFS, ICP-OES, XRD, in situ DRIFTS, catalytic reactivity, and DFT calculation results (PDF)
