# Supplementary material for: Maximizing Active Fe Species in ZSM-5 Zeolite Using Organic-Template-Free Synthesis for Efficient Selective Methane Oxidation
Source: J Am Chem Soc. 2023 Feb 14;145(10):5888–98. doi: 10.1021/jacs.2c13351 (PMC10021013; doi:10.1021/jacs.2c13351)
Supplement: Supplementary file 1 — ja2c13351_si_001.pdf [file ja2c13351_si_001.pdf]

Supporting Information

Maximizing Active Fe Species in ZSM-5 Zeolite Using Organic-Template-Free Synthesis for Efficient Selective Methane Oxidation

Qingpeng Cheng[1,2], Guanna Li[3,4], Xueli Yao[2], Lirong Zheng[5], Junhu Wang[6], Abdul-Hamid Emwas[7], Pedro Castaño[2], Javier Ruiz-Martínez[2], Yu Han[1,2*]

[1] King Abdullah University of Science and Technology (KAUST), Physical Sciences and Engineering Division, Advanced Membranes and Porous Materials (AMPM) Center, Thuwal 23955-6900, Saudi Arabia

[2] KAUST, KAUST Catalysis Center (KCC), Thuwal 23955-6900, Saudi Arabia

[3] Biobased Chemistry and Technology, Wageningen University & Research, Bornse Weilanden 9, Wageningen, 6708WG, The Netherlands

[4] Laboratory of Organic Chemistry, Wageningen University & Research, Stippeneng 4, Wageningen, 6708WE, The Netherlands

[5] Beijing Synchrotron Radiation Facility, Institute of High Energy Physics, Chinese Academy of Sciences, Beijing, 100049 China

[6] Center for Advanced Mössbauer Spectroscopy, Dalian Institute of Chemical Physics, Chinese Academy of Sciences, Dalian, Liaoning, 116023 China

[7] Imaging and Characterization Core Lab, King Abdullah University of Science and Technology, Thuwal 23955-6900, Saudi Arabia

Materials and Methods

Catalyst preparation

Synthesis of Fe-HZ5-TF. Fe-HZ5-TF was synthesized using a previously reported seeded growth template-free method with slight modifications. This process included two steps: the synthesis of ZSM-5 seeds and the synthesis of Fe-ZSM-5.

In the first step, 15 g of colloidal silica (Ludox HS40) was dissolved in 7 mL of a NaOH solution (1 M) under stirring at 100 °C for 1 h. A separate solution was prepared by dissolving 0.45 g of sodium aluminate in 7 mL of a NaOH solution (1 M). The two solutions were mixed to obtain a synthetic aluminosilicate gel with a molar composition of $4Na_2O$:$1Al_2O_3$:$36SiO_2$:$460H_2O$. The gel was stirred vigorously at 100 °C for 2 h and transferred to a stainless-steel autoclave for crystallization at 180 °C for 48 h. The obtained crystals were collected by filtration, washed with deionized water, and dried at 100 °C to produce the ZSM-5 seeds.

In the second step, a synthetic aluminosilicate gel was prepared using the same method as in the first step. Subsequently, 1.5 mL of an aqueous solution containing 0.165 g of iron oxalate (Fe loading: 0.40 wt% in the final product) and 0.06 g of seeds were added sequentially to the gel. After stirring for 30 min, the mixture was transferred to an autoclave for crystallization at 180 °C for 48 h. The obtained crystals were collected by filtration, washed with deionized water, and dried at 80 °C to obtain the as-synthesized Fe-Z5-TF. Finally, Fe-Z5-TF was converted to its H-form (Fe-HZ5-TF) via ion exchange with ammonium nitrate followed by calcination in air at 550 °C for 6 h. The Fe loading in Fe-HZ5-TF could be tuned by varying the amount of Fe precursor while keeping the other conditions unchanged.

Synthesis of Fe-HZ5-C. In the synthesis of Fe-HZ5-C (Si/Al = 33.5; Al/Fe = 10.8), 0.027g of iron oxalate was added to a solution containing 9.69 g of an aqueous tetrapropylammonium hydroxide solution (20 wt%), 5.6 g of tetraethyl orthosilicate, 1.0 g of urea, 0.33 g of $Al(NO_3)_3\cdot 9H_2O$, and 0.05 g of isopropyl alcohol. The resulting solution was transferred to an autoclave for crystallization at 180 °C for 48 h. The obtained crystals were collected by filtration, washed three times with deionized water, and dried at 80 °C to obtain the as-synthesized Fe-Z5-C. Fe-Z5-C was also converted to its H-form (Fe-HZ5-C) via ion exchange with ammonium nitrate,

followed by calcination in air at 550 °C for 6 h. The Al or Fe loading in Fe-HZ5-C can be tuned by varying the amount of $Al(NO_3)_3 \cdot 9H_2O$ or iron oxalate in the synthetic gel, while keeping the other conditions unchanged.

Synthesis of ^{57}Fe-ZSM-5. Four Fe-ZSM-5 samples were prepared with an iron isotope and subject to Mössbauer spectroscopic analysis: ^{57}Fe-Z5-C, ^{57}Fe-Z5-TF, ^{57}Fe-HZ5-C, and ^{57}Fe-HZ5-TF. They were synthesized using the same processes described above, except that $^{57}FeC_2O_4$ was used as the Fe precursor.

Catalyst characterization

Powder XRD (Bruker D8 Advance) was performed at 40 kV and 40 mA using CuKα radiation (λ = 1.5418 Å). Diffuse reflectance UV–Vis spectra were recorded at a scanning rate of 200 nm/min on a Varian Cary 5000 spectrophotometer equipped with a diffuse reflectance accessory (Praying Mantis, Harrick). XAS (Fe K-edge) were obtained at the 1W1B station at the Beijing Synchrotron Radiation Facility (BSRF). The storage rings at the BSRF were operated at 2.5 GeV with a maximum current of 250 mA. With a Si (111) double-crystal monochromator, the data were collected in transmission mode using an ionization chamber for Fe foil and Fe_2O_3 and in fluorescence excitation mode using a Lytle detector for Fe-containing zeolites. The XANES data were analyzed using ATHENA software, while the Fourier-transformed EXAFS data were analyzed using ARTEMIS software and the wavelet-transformed EXAFS data were analyzed using MATLAB software. The Mössbauer spectra were acquired from self-supporting pellets of ^{57}Fe-containing zeolites using a Topologic 500A spectrometer (Topologic Systems). The source of the γ rays was ^{57}Co in a rhodium matrix, and α-Fe foil was used as a reference. The spectra were collected at room temperature and fitted using MossWinn software. The composition of the catalysts was determined using inductively coupled plasma optical emission spectrometry (ICP-OES, Varian 7200-ES). Electron paramagnetic resonance measurements were performed at room temperature using a Bruker EMX-10/12 EPR spectrometer operated in the X-band frequency using the following parameters: microwave frequency of 9.8 GZ, microwave power of 20 mW, modulation frequency of 100 kHz, and a 10 dB attenuator. The in situ diffuse reflectance Fourier transform infrared (FTIR) spectra were recorded on a NICOLET 6700 IR spectrometer equipped with a liquid nitrogen-cooled mercury cadmium telluride detector and high-temperature reaction chamber (SeZn windows). The catalysts were packed into the reaction chamber and pretreated in a He stream (20 mL min^{-1}) at 400 °C for 30 min. After cooling to 75 °C for background

spectra collection, CH_4 and H_2O_2 were introduced into the gas stream while the time-resolved spectra were collected.

Catalytic selective oxidation of methane

The selective CH_4 oxidation reactions were carried out in a stainless-steel autoclave reactor containing a Teflon liner vessel (working volume, 100 mL). In a typical run, the reactor was first charged with 27 mg of catalyst, 10 mL of H_2O_2 aqueous solution (0.5 M), then purged three times with 10 bar CH_4, and finally pressurized to 30.5 bar with CH_4. The reactor was heated to 75 ^{o}C with a stirring speed of 500 rpm. After 25 min of the reaction, the reactor was cooled to < 5 °C using an ice-water mixture before product analysis. The gas-phase products were collected using a gas-sampling bag and analyzed using gas chromatography (GC) with a flame ionization detector (FID). Considering the instability of peroxides, the liquid phase products were separated from the catalyst by centrifugation, stored at 0 °C, and analyzed using ^{1}H-nuclear magnetic resonance (NMR, Bruker AVANCE III 600 MHz) spectroscopy within several hours. A 490 μL aliquot of the products was mixed with 110 μL of D_2O containing 1.67 ppm of dimethyl sulfoxide (DMSO) as the internal standard. The ^{1}H chemical shifts of methyl protons in DMSO, methyl protons in CH_3OH, methyl protons in CH_3OOH, methylene protons in $HOCH_2OOH$ and methine proton in HCOOH are 2.7, 3.4, 3.8, 5.1 and 8.3 ppm, respectively.

DFT calculations

All spin-polarized DFT calculations were conducted using the Vienna Ab initio Simulation Package (VASP, version 5.4.4).[1-2] The Perdew−Burke−Ernzerhof (PBE) functional based on the generalized gradient approximation was chosen to account for the exchange–correlation energy.[3] A plane-wave basis set in combination with the projected augmented wave (PAW) method was used to describe the valence electrons and the valence−core interactions, respectively.[4] The kinetic energy cut-off for the plane-wave basis set was 500 eV. Gaussian smearing of the population of partial occupancies with a width of 0.05 eV was used during the iterative diagonalization of the Kohn-Sham Hamiltonian. The threshold for energy convergence for each iteration was set to 10^{-5} eV. The geometries were assumed to have converged when the force on each atom was less than 0.05 eV/Å. Considering the large unit cell, Brillouin zone sampling was simulated using k-point meshes of 1 × 1 × 2.

The Van der Waals (vdW) interactions were included using Grimme's DFT-D3(BJ) method as implemented in VASP.[5]

The orthogonal MFI unit cell with lattice parameters of a = 20.09 Å, b = 19.93 Å, and c = 13.42 Å, as optimized using DFT with an all-silica MFI periodic model, was used for all calculations. The optimized unit cell parameters agreed well with experimental data for calcined ZSM-5.[6] One or two framework Si^{4+} ions in the MFI unit cell were substituted by two Al^{3+} to compensate for the positive charge of the extra-framework cationic iron complexes. The monovalent iron complex was stabilized by one Al^{3+} at the T11 site located in the 10-membered ring of a straight channel. The six-membered ring of the β-site was considered to accommodate the bivalent Fe complex, with an Al^{3+} pair at T10 and T11.

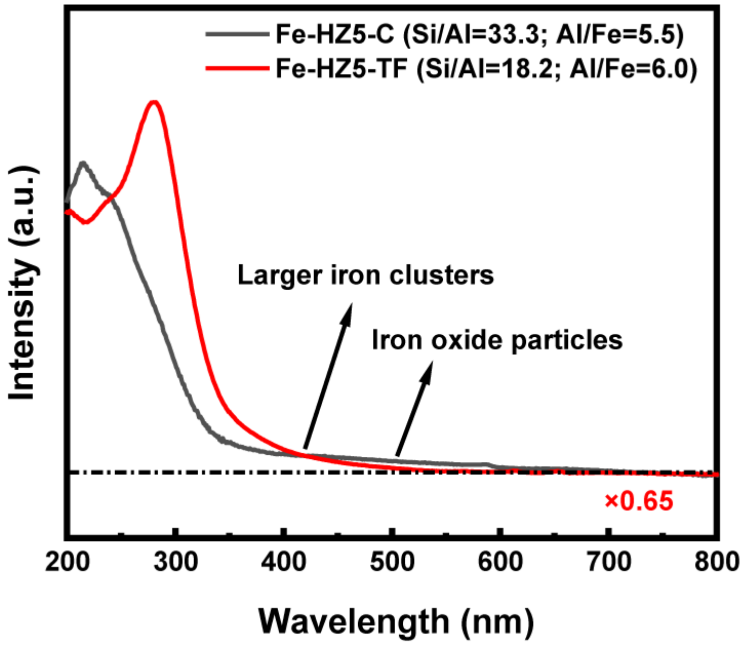

Figure S1. DR UV-Vis spectra of Fe-ZSM-5 samples synthesized using the conventional TPA-templating (Si/Al=33.3; Al/Fe=5.5) and template-free (Si/Al=18.2; Al/Fe=6.0) methods.

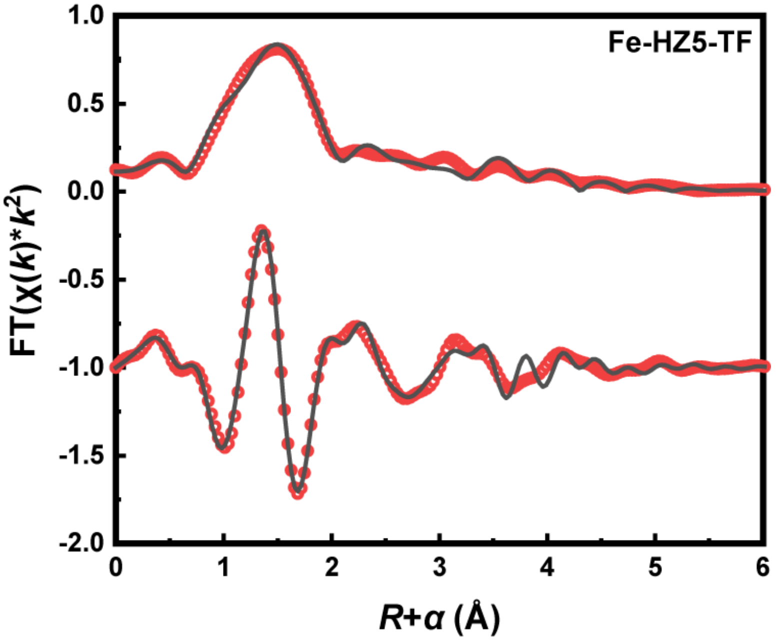

Figure S2. Fitting results for the Fe K-edge EXAFS of Fe-HZ5-TF using the four paths Fe-O1, Fe-O2, Fe-Al, and Fe-Fe, shown in R space (FT magnitude and imaginary component).

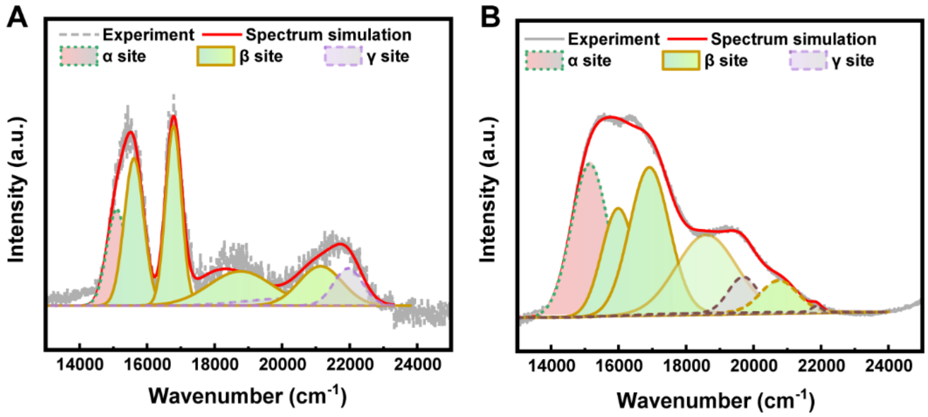

C

Samples	Si/Al	Co/Al	Single Al (%)	Al pairs (%)	Al pairs distribution (%)		
					α	β	γ
Co-Z5-C	36.5	0.05	90.2	9.8	15.5	78.6	5.8
Co-Z5-TF	17.2	0.14	71.2	28.8	25.5	69.3	5.2

Figure S3. (**A** and **B**) DR UV-Vis spectra of dehydrated Co-Z5-C (A) and dehydrated Co-Z5-TF (B). The spectra were deconvoluted using Gaussian bands to determine the proportions of different Al pair sites (α, β, and γ sites), according to an established method.[7-8] The α sites correspond to the 6MR on the wall of the straight channel; the β sites correspond to the 6MR at the intersection of the straight and sinusoidal channels; and the γ sites are the cavities formed by the 5 and 6MR in the sinusoidal channel. (**C**) Chemical composition of Co-Z5-C and Co-Z5-TF. The Si, Al, and Co content was determined using ICP-OES. The amount of Al pairs was determined from the Co content based on their one-to-one correspondence (each Co ion is immobilized by an Al pair, while there are no free Co ions).

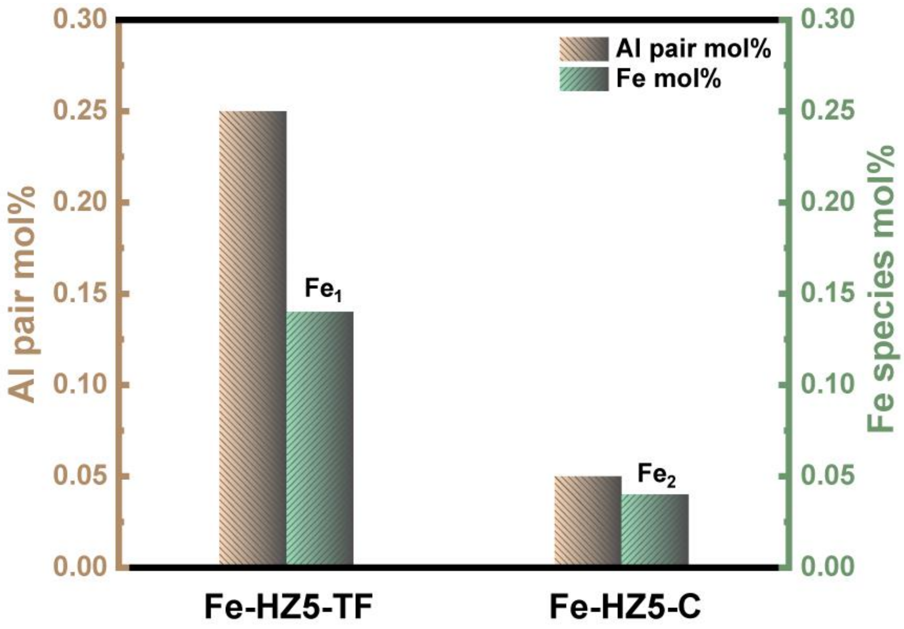

Figure S4. Amount of Al pairs, mononuclear Fe (Fe_1), and binuclear Fe (Fe_2) in Fe-HZ5-TF and Fe-HZ5-C expressed in mole percentage relative to all elements.

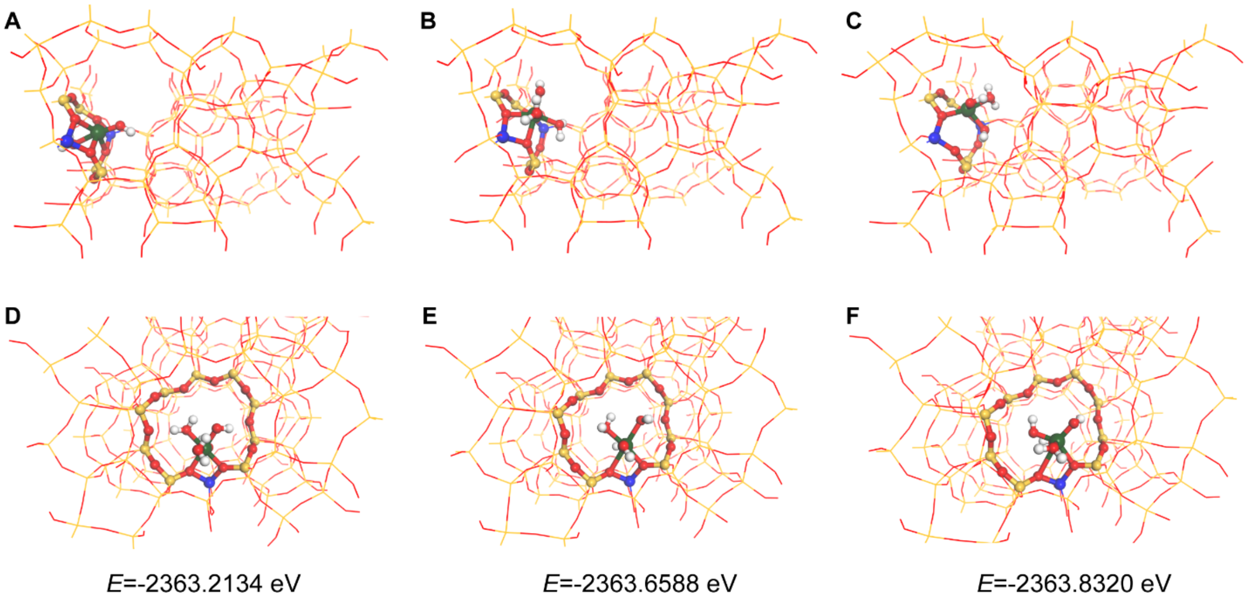

Figure S5. (**A-C**) DFT-optimized structure of mononuclear Fe, $[(H_2O)_{n=1,2,3}\text{-Fe(III)-OH}]^{2+}$, bound to an Al pair in the 6MR at the β site (the intersection of the straight and sinusoidal channels) of ZSM-5. The Fe center is 6-coordinated in an octahedral geometry. Thus, in (A), (B), and (C), where *n* is 1, 2, and 3, respectively, the Fe center is bound to 4, 3, and 2 framework oxygen (O_f), respectively. (**D-E**) DFT-optimized structure of mononuclear Fe, $[(H_2O)_2\text{-Fe(III)-(OH)}_2]^+$, bound to a single Al site in the 10MR of the ZSM-5 straight channel, with different ligands allocated to the octahedral apical sites: (D) one OH and one H_2O, (E) two OH, and (F) two H_2O. The *E* value represents the total energy of each model calculated using DFT. Yellow spheres: Si atoms; red spheres: O atoms; blue spheres: Al atoms; green spheres: Fe atoms.

Note: Spin-polarized periodic DFT calculations were conducted using the Vienna *Ab initio* Simulation Package (VASP) to investigate the binding of octahedrally coordinated mononuclear Fe with Al pairs in the ZSM-5 framework. Given that Al pairs are primarily located at the β site (see Figure S3), three types of octahedral mononuclear $[(H_2O)_n\text{-Fe(III)-OH}]^{2+}$ complex (n = 1, 2 , or 3) were placed in the 6MR of the β site for the calculations, where they interacted with an Al

pair through 4, 3, and 2 framework oxygen (O_f) atoms, respectively (Figure S5, A-C). Of the three optimized models, the $[(H_2O)_2\text{-Fe(III)-OH}]^{2+}$ bound to three zeolite O_f atoms (Figure S5B and Table S1), containing one hydroxyl as a charge compensator and two coordinated H_2O molecules, exhibited the closest agreement with the EXAFS fitting results in terms of the Fe–O bond lengths (Table 2). Therefore, the mononuclear Fe species in ZSM-5 was determined to be in the form of $[(H_2O)_2\text{-Fe(III)-OH}]^{2+}$ bound to three zeolite O_f atoms.

In addition, the binding of mononuclear Fe with a single Al site was also calculated using a model in which $[(H_2O)_2\text{-Fe(III)-(OH)}_2]^{+}$ was located in the 10MR straight channel of ZSM-5. Three types of ligand configuration were investigated (Figures S5D-S5F). The results revealed that, of the three ligand configurations, the structure with two H_2O molecules located at the octahedral apical sites (Figure S5F) was the most energetically stable. However, the optimized structure of this configuration contained three sets of Fe–O bond lengths (1.84, 2.15, and 2.93 Å) (Table S1). These structural properties were inconsistent with the EXAFS fitting results (two sets of Fe–O bond lengths: 1.86 and 2.03 Å). As such, mononuclear Fe binding to a single Al site was ruled out.

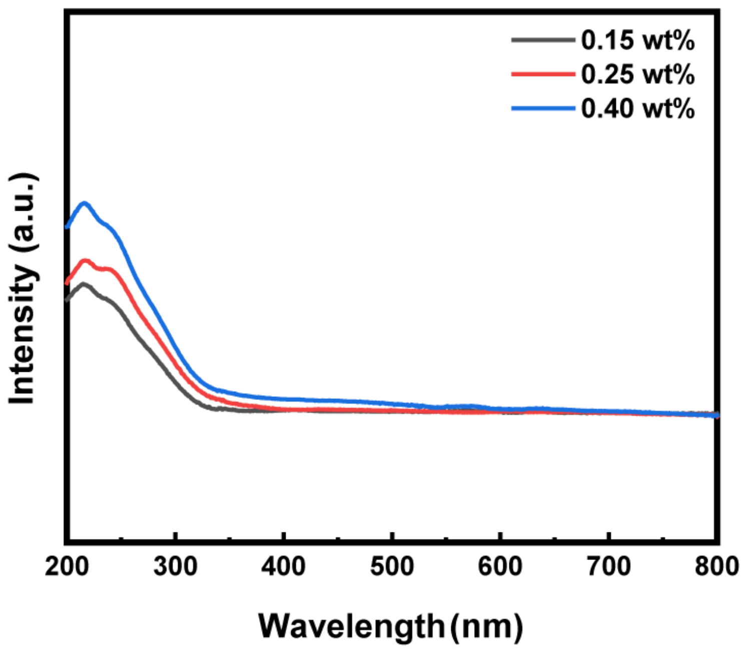

Figure S6. DR UV-Vis spectra for Fe-HZ5-C samples with a fixed Si/Al ratio of ~35 but different Fe loadings (0.15, 0.25, and 0.40 wt%).

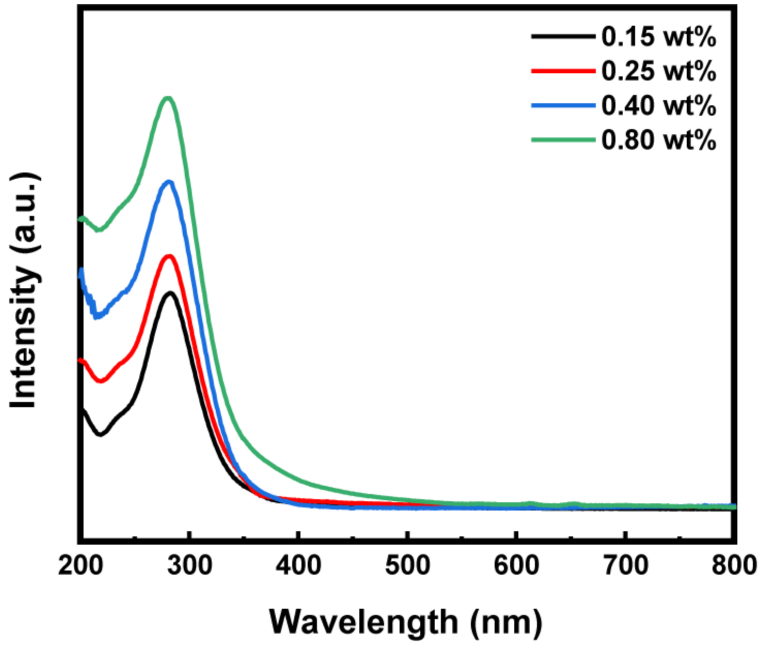

Figure S7. DR UV-Vis spectra for Fe-HZ5-TF samples with a fixed Si/Al ratio of ~18 but different Fe loadings (0.15, 0.25, 0.40, and 0.80 wt%).

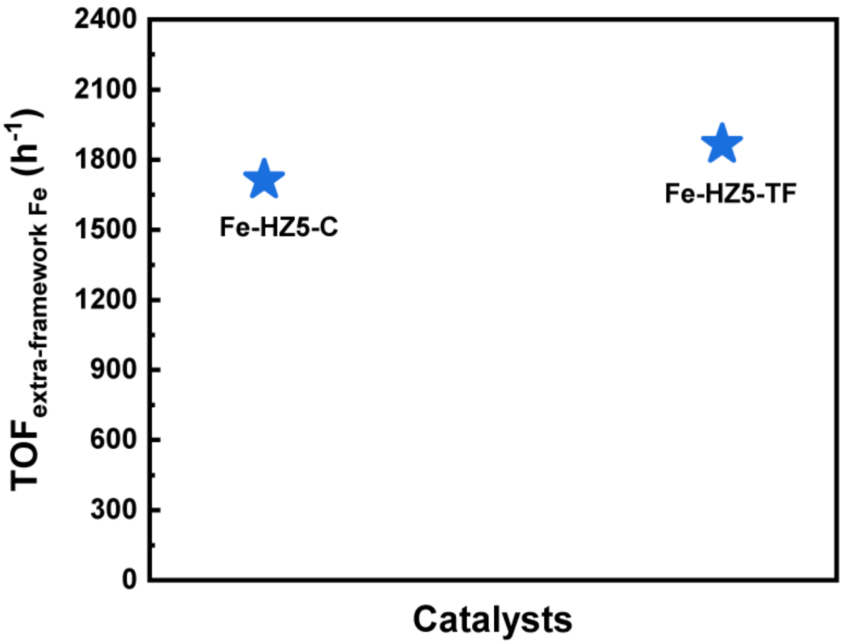

Figure S8. TOF for Fe-HZ5-C (Fe loading: 0.25 wt%) and Fe-HZ5-TF (Fe loading: 0.40 wt%) for the selective oxidation of methane at 75 °C, calculated based on the amount of active (i.e., extra-framework) Fe species.

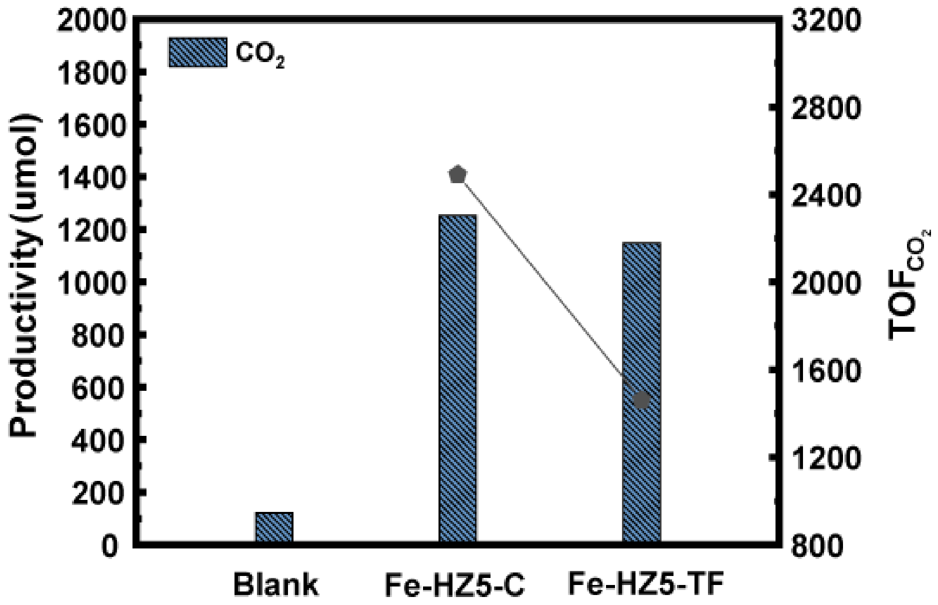

Figure S9. HCOOH oxidation experiments. Reaction conditions: 27 mg catalyst, 10.0 mL HCOOH aqueous solution (0.3 M) containing 0.5 M H_2O_2, 30.5 bar N_2, 75 °C, and 25 min.

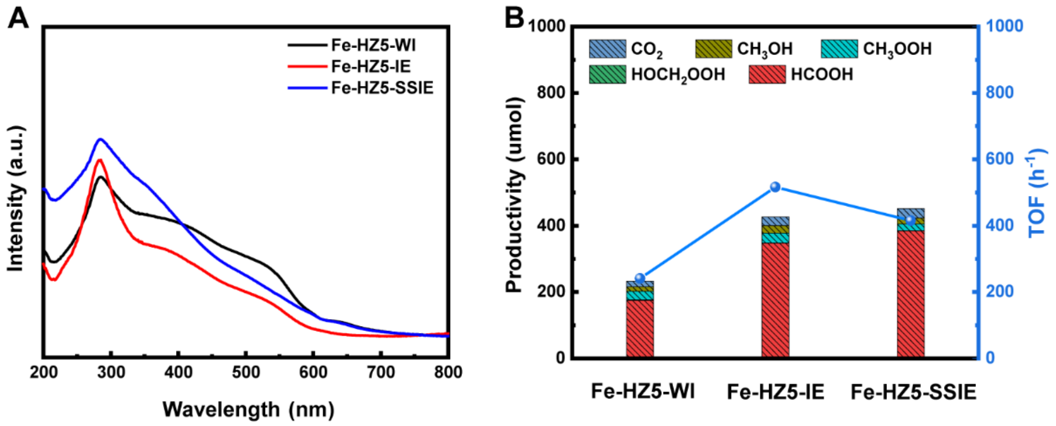

Figure S10. (**A**) DR UV-Vis spectra and (**B**) catalytic performance of Fe-HZSM-5 samples prepared using different post-synthesis methods, including wet impregnation (WI),[9] aqueous-phase ion exchange (IE),[9] and solid-state ion exchange (SSIE).[10] The Fe loadings in the three samples were 0.48, 0.41, and 0.54 wt%.

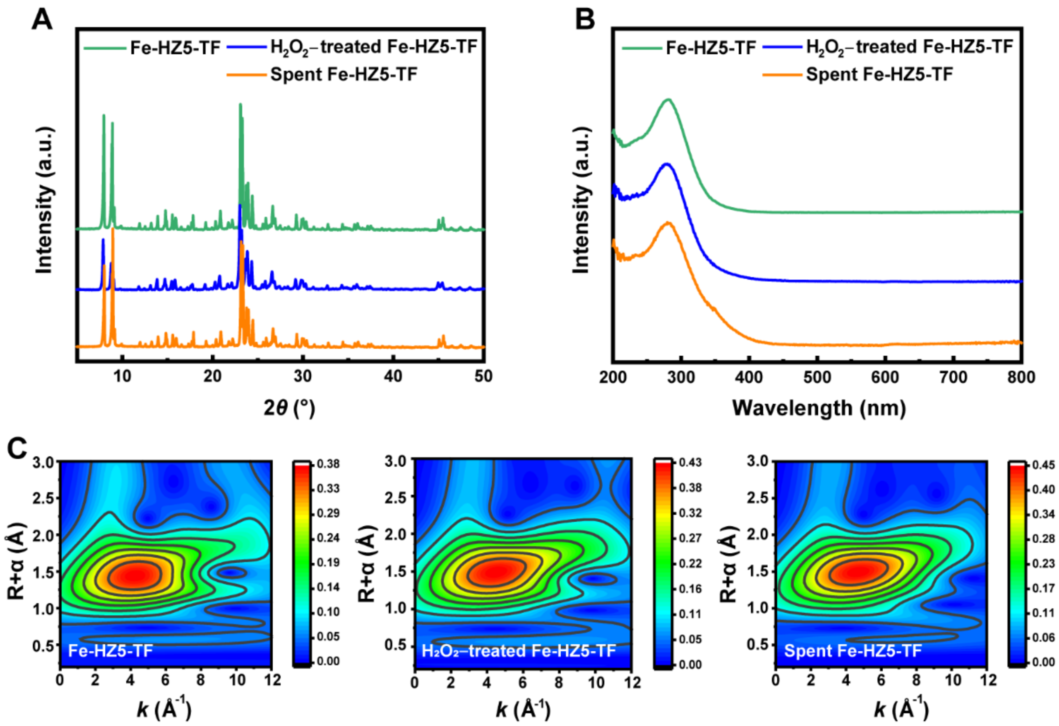

Figure S11. (**A**) Powder XRD patterns. (**B**) DR UV-Vis spectra. (**C**) WT-EXAFS contour plots for Fe-HZ5-TF in different states (pristine, H_2O_2-treated, and spent). The results indicate that, following the addition of H_2O_2 or during the catalytic reaction, the mononuclear Fe species remained isolated and did not agglomerate to form Fe_xO_y clusters or particles.

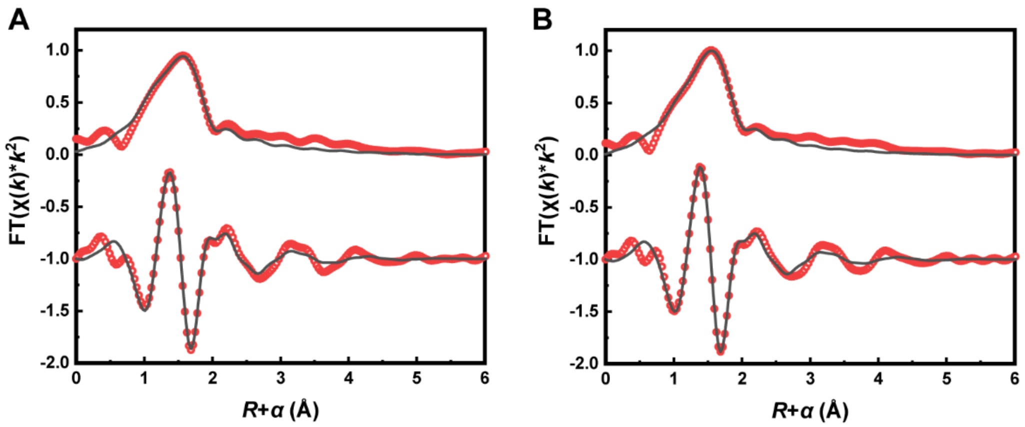

Figure S12. (**A** and **B**) Fitting results for the Fe K-edge EXAFS spectra of H_2O_2-treated (A) and spent Fe-HZ5-TF (B) shown in R space (FT magnitude and imaginary component). See Table S5 for the average fine-structure parameters.

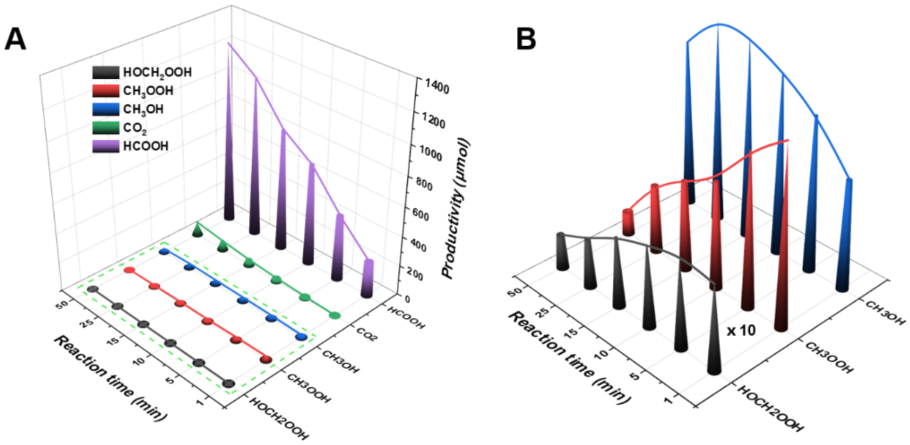

Figure S13. (**A**) Evolution of various products during the selective oxidation of methane on Fe-HZ5-TF. (**B**) Magnified view of the CH_3OH, CH_3OOH, and $HOCH_2OOH$ profiles in (A).

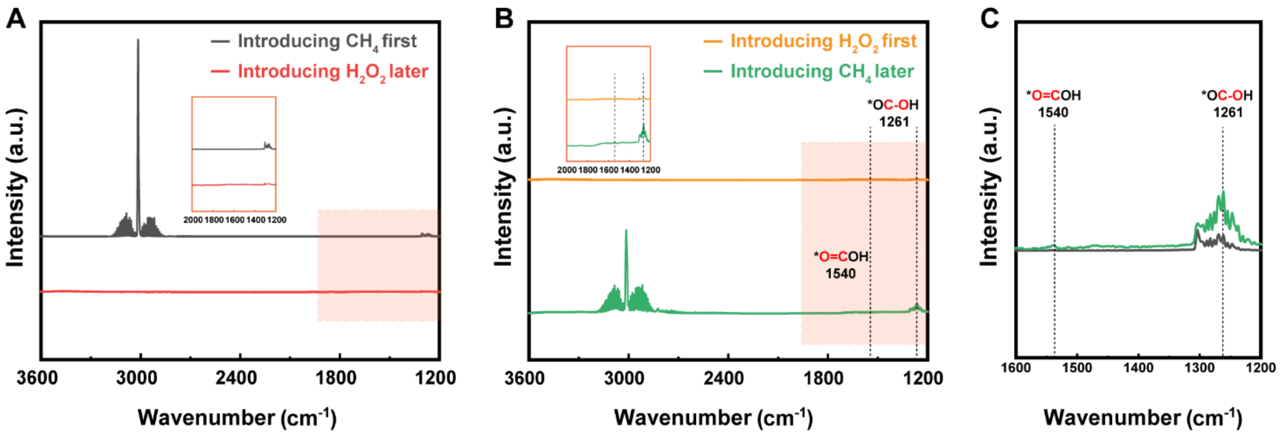

Figure S14. In situ DRIFTS results of introducing CH_4 and H_2O_2 stepwise into an in situ cell at 75 °C. (**A**) Introducing CH_4 first (black), followed by H_2O_2 (red). (**B**) Introducing H_2O_2 first (orange), followed by CH_4 (green). In each panel, the inset is an enlarged view of the highlighted area. (**C**) Enlarged black and green spectra for comparison. Compared to the black spectrum that only exhibits peaks related to CH_4, the green spectrum has additional peaks associated with oxygenates (e.g., at 1261 cm^{-1} and 1540 cm^{-1}, as highlighted with the dash line)

Note: The experimental operation of (A) was as follows. The Fe-HZ5-TF powder was packed into the in situ cell and degassed at 400 °C for 0.5 h. Then, the temperature of the cell was reduced to 75 °C under a helium gas flows. Record a spectrum after switching the gas flow to CH_4 and stabilizing for 10 min. Subsequently, the cell was purged with helium to remove residual CH_4, followed by the introduction of H_2O_2 using a helium carrier gas. After 10 min, record another spectrum. The other experiment (B) was performed by following the same procedure except that the order of the introduction of two reactants was reversed.

Table S1. Calculated Fe–O bond lengths from the six models shown in Figure S5.[a]

Bond	Bond Length (Å) \ Model	Al pairs 5A	Al pairs 5B	Al pairs 5C	Single Al 5D	Single Al 5E	Single Al 5F
Fe-O	$Fe\text{-}O_f$	2.07	2.07	1.98	2.03	2.04	2.16
		2.14	2.26	2.01	2.11	2.42	2.93
		2.14	2.29	/	/	/	/
	Fe-OH	2.38	/	/	/	/	/
		1.79	1.82	1.76	1.79	1.79	1.81
		/	/	/	1.85	1.80	1.86
	$Fe\text{-}H_2O$	2.18	1.99	2.03	1.98	2.05	2.12
		/	2.17	2.07	2.08	2.29	2.16
		/	/	/	/	/	/
	Average	1.93_2	1.90_2	1.87_2	1.82_2	1.80_2	1.84_2
		2.21_4	2.20_4	2.04_3	2.05_4	2.20_4	2.15_3
		/	/	/	/	/	2.93_1

[a] 5A−5F correspond to the six DFT-optimized structural models in Figure S5. The subscripts represent the average Fe-O coordination number obtained from the calculations.

Table S2. Catalytic performance of Fe-HZ5-TF compared with various catalysts reported for the selective oxidation of methane under mild conditions.

#	Catalysts	**Reaction conditions**					**C1 oxygenate productivity (μmol)**					**Activity**		Ref.
		P (bar)	T (°C)	Cat. (mg)	H_2O_2 (M)	t (min)	CO_2	CH_3OH	CH_3OOH	$HOCH_2OOH$	HCOOH	TOF (h^{-1})	Yield ($mmolg_{cat}^{-1}h^{-1}$)	
1	FeN_4/GN	20.0	25	50	5.0	600	7.66	5.6	41.4	34.46	35.54	0.47	0.25	11
2	FeOx/TiO_2	1.0	25	10	0.0008	180	0.12	6.7	/	/	0	13.3	0.3	12
3	Rh/CeO_2	5.0	50	10	1.0	60	1.5	27.3	8.5	/	/	126.7	3.6	13
4	Fe/ZSM-5	30.0	50	300	0.5	30	31	177	248	191	431	268.7	7.2	14
5	UiO-66(2.5TFA)-Fe	30.0	50	25	0.3	60	2.6	6.5	10.0	25.7	77.0	12.4	4.9	15
6	Cr/TiO_2	30.0	50	10	0.5	60	/	3.4	17.5	20.8	2.2	21.6	4.4	16
7	Fe/ZSM-5	30.5	50	50	~0.3	60	138	49	12	/	796	258.5	19.9	9
8	Fe/ZSM-5	30.5	50	27	0.5	30	15.8	20.1	3	/	158.5	153.7	14.6	17
9	Fe/ZSM-5	30.0	50	216	1.0	30	5.1	10	/	/	84	324.0	0.9	18
10	PdAu/CNTs	33.0	50	30		30	/	4.17	0.26	2	1.27	2.2	0.5	19
11	Au-Pd colloid	30.0	50	1.0	1.0	30	1.1	3.3	11.8	/	0.6	5.1	33.6	20
12	Fe/ZSM-5	30.0	50	20	0.5	30	3.6	14.5	4.8	/	147	3398	17.0	21
13	Ga,Fe-MFI	30.6	55	28	0.5		25.0	36.0	7.7	/	648.1	159.7	51.2	22
14	MIL-53(Fe,Al)	30.5	60	/	0.5	240	/	/	/	/	/	90	7.8	23
15	Rh/ZrO_2	30.0	70	30	0.5	30	0.3	0.9	0.2	/	/	1.57	0.05	24
16	Pd/ZSM-5	30.0	70	28	0.5	30	18.4	12.8	40.2	/	240.5	23705.2	22.3	25
17	AuPd@ZSM-5	30.0	70	27	0.1	30	/	39.2	/	/	2.7	/	3.1	26
18	Fe-MOR	30.0	80	30	0.5	60	37*	25*	25*	50*	75*	78.9*	7.1*	27
19	Fe-MFI	30.0	80	50	2.7	120	0	50	/	/	1890	23.0	19.4	28
20	AuPd/TiO_2	30.5	90	10	0.5	30	1.08	1.84	6.39	/	0	25.2	1.9	29
21	Fe-HZ5-TF (0.4 wt% Fe)	30.5	75	27	0.5	25	69.8	29.6	8.7	1.4	1120.9	1566.1	109.4	This work
22	Fe-HZ5-TF (0.4 wt% Fe)	30.5	50	27	0.5	25	10.2	34.2	24.7	3.2	594.7	849.0	59.3	
23	Fe-HZ5-C (0.25 wt% Fe)	30.5	75	27	0.5	25	35.6	15.7	7.6	0.6	354.2	821.9	36.8	
24	Fe-HZ5-C (0.25 wt% Fe)	30.5	50	27	0.5	25	15.9	17.3	9.9	1.5	182.5	451.0	20.2	

*Estimated from the graphs in the literature.

Table S3. Catalytic performance for selective oxidation of ethane. [a]

Catalysts	Products (umol)							$Sel(C_2)$	TOF (h^{-1})	Yield (mmol $g_{cat}^{-1}h^{-1}$)
	CH_3CH_2OH	CH_3COOH	CO_2	CH_3OH	CH_3OOH	$HOCH_3OOH$	HCOOH			
Fe-HZ5-TF	192.9	1615.1	65.5	107.5	27.2	1.7	792.4	64.1	2382.1	166.3

[a] Reaction conditions: 40 mg catalyst, 10.0 mL H_2O_2 aqueous solution (1.0 M), 20 bar C_2H_6, 75 °C, and 25 min.

Table S4. Catalytic performance for selective oxidation of propane.[a]

Catalysts	Products (umol)												Sel(C_2+C_3)	TOF (h^{-1})	Yield (mmol $g_{cat}^{-1}h^{-1}$)
	$CH_3CHOHCH_3$	CH_3CH_3CHO	CH_3CH_3COOH	CH_3COCH_3	$CH_3CH_3CH_2OH$	CH_3CH_2OH	CH_3COOH	CO_2	CH_3OH	CH_3OOH	$HOCH_3OOH$	$HCOOH$			
Fe-HZ5-TF	18.1	116.8	337.4	795.7	28.0	100.3	311.4	136.9	40.6	0	1.1	449.7	73.1	2007.2	140.2

[a] Reaction conditions: 40 mg catalyst, 10.0 mL H_2O_2 aqueous solution (1.0 M), 5.0 bar C_3H_8, 15.0 bar N_2, 75 °C, and 25 min.

Table S5. Fe K-edge EXAFS curve fitting parameters.[a]

Samples	Path	R (Å)	CN	Δ^2(*100Å^2)	ΔE_0 (eV)	R-factor
H_2O_2-treated Fe-HZ5-TF	Fe-O1	1.86 (0.03)	2.0 (0.5)	0.6 (0.2)	4 (2)	0.006
	Fe-O2	2.05 (0.02)	4.5[b]	0.7 (0.3)		
Spent Fe-HZ5-TF	Fe-O1	1.86 (0.03)	1.8 (0.6)	0.8 (0.3)	5 (2)	0.005
	Fe-O2	2.04 (0.02)	4.8[b]	0.8 (0.3)		

[a] R, distance between the absorber and backscatter atoms; CN, coordination number; δ^2, Debye-Waller factor to account for both thermal and structural disorder; ΔE_0, inner potential correction; R-factor indicates the goodness of the fit. [b] Restrained value.

References

(1) Kresse, G.; Furthmüller, J., Efficient iterative schemes for *ab initio* total-energy calculations using a plane-wave basis set. *Phys. Rev. B* **1996,** *54* (16), 11169-11186.

(2) Kresse, G.; Joubert, D., From ultrasoft pseudopotentials to the projector augmented-wave method. *Phys. Rev. B* **1999,** *59* (3), 1758-1775.

(3) Perdew, J. P.; Burke, K.; Ernzerhof, M., Generalized gradient approximation made simple. *Phys. Rev. Lett.* **1996,** *77* (18), 3865-3868.

(4) Blöchl, P. E., Projector augmented-wave method. *Phys. Rev. B* **1994,** *50* (24), 17953-17979.

(5) Grimme, S.; Ehrlich, S.; Goerigk, L., Effect of the damping function in dispersion corrected density functional theory. *J. Comput. Chem.* **2011,** *32* (7), 1456-1465.

(6) van Koningsveld, H.; Jansen, J. C.; van Bekkum, H., The monoclinic framework structure of zeolite H-ZSM-5. Comparison with the orthorhombic framework of as-synthesized ZSM-5. *Zeolites* **1990,** *10* (4), 235-242.

(7) Dedecek, J.; Balgová, V.; Pashkova, V.; Klein, P.; Wichterlová, B., Synthesis of ZSM-5 zeolites with defined distribution of Al atoms in the framework and multinuclear MAS NMR analysis of the control of Al distribution. *Chem. Mater.* **2012,** *24* (16), 3231-3239.

(8) Dědeček, J.; Tabor, E.; Sklenak, S., Tuning the aluminum distribution in zeolites to increase their performance in acid-catalyzed reactions. *ChemSusChem* **2019,** *12* (3), 556-576.

(9) Kim, M. S.; Park, K. H.; Cho, S. J.; Park, E. D., Partial oxidation of methane with hydrogen peroxide over Fe-ZSM-5 catalyst. *Catal. Today* **2021,** *376*, 113-118.

(10) Hammond, C.; Forde, M. M.; Ab Rahim, M. H.; Thetford, A.; He, Q.; Jenkins, R. L.; Dimitratos, N.; Lopez-Sanchez, J. A.; Dummer, N. F.; Murphy, D. M.; Carley, A. F.; Taylor, S. H.; Willock, D. J.; Stangland, E. E.; Kang, J.; Hagen, H.; Kiely, C. J.; Hutchings, G. J., Direct catalytic conversion of methane to methanol in an aqueous medium by using copper-promoted Fe-ZSM-5. *Angew. Chem. Int. Ed.* **2012,** *124* (21), 5219-5223.

(11) Cui, X.; Li, H.; Wang, Y.; Hu, Y.; Hua, L.; Li, H.; Han, X.; Liu, Q.; Yang, F.; He, L.; Chen, X.; Li, Q.; Xiao, J.; Deng, D.; Bao, X., Room-temperature methane conversion by graphene-confined single iron atoms. *Chem* **2018,** *4* (8), 1902-1910.
(12) Xie, J.; Jin, R.; Li, A.; Bi, Y.; Ruan, Q.; Deng, Y.; Zhang, Y.; Yao, S.; Sankar, G.; Ma, D., Highly selective oxidation of methane to methanol at ambient conditions by titanium dioxide-supported iron species. *Nat. Catal.* **2018,** *1* (11), 889-896.
(13) Bai, S.; Liu, F.; Huang, B.; Li, F.; Lin, H.; Wu, T.; Sun, M.; Wu, J.; Shao, Q.; Xu, Y., High-efficiency direct methane conversion to oxygenates on a cerium dioxide nanowires supported rhodium single-atom catalyst. *Nat. Commun.* **2020,** *11* (1), 1-9.
(14) Yu, T.; Li, Z.; Jones, W.; Liu, Y.; He, Q.; Song, W.; Du, P.; Yang, B.; An, H.; Farmer, D. M., Identifying key mononuclear Fe species for low-temperature methane oxidation. *Chem. Sci.* **2021,** *12* (9), 3152-3160.
(15) Zhao, W.; Shi, Y.; Jiang, Y.; Zhang, X.; Long, C.; An, P.; Zhu, Y.; Shao, S.; Yan, Z.; Li, G., Fe-O clusters anchored on nodes of metal-organic frameworks for direct methane oxidation. *Angew. Chem. Int. Ed.* **2021,** *133* (11), 5875-5879.
(16) Shen, Q.; Cao, C.; Huang, R.; Zhu, L.; Zhou, X.; Zhang, Q.; Gu, L.; Song, W., Single chromium atoms supported on titanium dioxide nanoparticles for synergic catalytic methane conversion under mild conditions. *Angew. Chem. Int. Ed.* **2020,** *59* (3), 1216-1219.
(17) Hammond, C.; Dimitratos, N.; Lopez-Sanchez, J. A.; Jenkins, R. L.; Whiting, G.; Kondrat, S. A.; ab Rahim, M. H.; Forde, M. M.; Thetford, A.; Hagen, H.; Stangland, E. E.; Moulijn, J. M.; Taylor, S. H.; Willock, D. J.; Hutchings, G. J., Aqueous-phase methane oxidation over Fe-MFI zeolites; promotion through isomorphous framework substitution. *ACS Catal.* **2013,** *3* (8), 1835-1844.
(18) Taran, O. P.; Yashnik, S. A.; Boltenkov, V. V.; Parkhomchuk, E. V.; Sashkina, K. A.; Ayusheev, A. B.; Babushkin, D. E.; Parmon, V. N., Formic acid production via methane peroxide oxidation over oxalic acid activated Fe-MFI catalysts. *Top. Catal.* **2019,** *62* (5), 491-507.

(19) He, Y.; Liang, J.; Imai, Y.; Ueda, K.; Li, H.; Yang, G.; Yoneyama, Y.; Tsubaki, N., Highly selective synthesis of methanol from methane over carbon materials supported Pd-Au nanoparticles under mild conditions. *Catal. Today* **2020,** *352*, 104-110.

(20) Agarwal, N.; Freakley, S. J.; McVicker, R. U.; Althahban, S. M.; Dimitratos, N.; He, Q.; Morgan, D. J.; Jenkins, R. L.; Willock, D. J.; Taylor, S. H., Aqueous Au-Pd colloids catalyze selective CH_4 oxidation to CH_3OH with O_2 under mild conditions. *Science* **2017,** *358* (6360), 223-227.

(21) Zhu, K.; Liang, S.; Cui, X.; Huang, R.; Wan, N.; Hua, L.; Li, H.; Chen, H.; Zhao, Z.; Hou, G., Highly efficient conversion of methane to formic acid under mild conditions at ZSM-5-confined Fe-sites. *Nano Energy* **2021,** *82*, 105718.

(22) Shahami, M.; Shantz, D. F., Zeolite acidity strongly influences hydrogen peroxide activation and oxygenate selectivity in the partial oxidation of methane over M, Fe-MFI (M: Ga, Al, B) zeolites. *Catal. Sci. Technol.* **2019,** *9* (11), 2945-2951.

(23) Osadchii, D. Y.; Olivos-Suarez, A. I.; Szécsényi, Á.; Li, G.; Nasalevich, M. A.; Dugulan, I. A.; Crespo, P. S.; Hensen, E. J.; Veber, S. L.; Fedin, M. V., Isolated Fe sites in metal organic frameworks catalyze the direct conversion of methane to methanol. *ACS Catal.* **2018,** *8* (6), 5542-5548.

(24) Kwon, Y.; Kim, T. Y.; Kwon, G.; Yi, J.; Lee, H., Selective activation of methane on single-atom catalyst of rhodium dispersed on zirconia for direct conversion. *J. Am. Chem. Soc.* **2017,** *139* (48), 17694-17699.

(25) Huang, W.; Zhang, S.; Tang, Y.; Li, Y.; Nguyen, L.; Li, Y.; Shan, J.; Xiao, D.; Gagne, R.; Frenkel, A. I., Low - temperature transformation of methane to methanol on Pd1O4 single sites anchored on the internal surface of microporous silicate. *Angew. Chem. Int. Ed.* **2016,** *128* (43), 13639-13643.

(26) Jin, Z.; Wang, L.; Zuidema, E.; Mondal, K.; Zhang, M.; Zhang, J.; Wang, C.; Meng, X.; Yang, H.; Mesters, C., Hydrophobic zeolite modification for in situ peroxide formation in methane oxidation to methanol. *Science* **2020,** *367* (6474), 193-197.

(27) Fang, Z.; Murayama, H.; Zhao, Q.; Liu, B.; Jiang, F.; Xu, Y.; Tokunaga, M.; Liu, X., Selective mild oxidation of methane to methanol or formic acid on Fe-MOR catalysts. *Catal. Sci. Technol.* **2019,** *9* (24), 6946-6956.

(28) Xiao, P.; Wang, Y.; Nishitoba, T.; Kondo, J. N.; Yokoi, T., Selective oxidation of methane to methanol with H_2O_2 over an Fe-MFI zeolite catalyst using sulfolane solvent. *Chem. Commun.* **2019,** *55* (20), 2896-2899.

(29) Ab Rahim, M. H.; Forde, M. M.; Jenkins, R. L.; Hammond, C.; He, Q.; Dimitratos, N.; Lopez‐Sanchez, J. A.; Carley, A. F.; Taylor, S. H.; Willock, D. J., Oxidation of methane to methanol with hydrogen peroxide using supported gold–palladium alloy nanoparticles. *Angew. Chem. Int. Ed.* **2013,** *52* (4), 1280-1284.
